# A randomized multi-arm open labelled comparative clinical trial report of Pankajakasthuri DiabetEaze powder, a novel polyherbal formulation on the nutritional management and glycemic control in type 2 diabetic and prediabetic patients

**DOI:** 10.1016/j.heliyon.2025.e42631

**Published:** 2025-02-13

**Authors:** Shan Sasidharan, Kasthuri Nair A, Lekshmi R, Arun Visakh Nair, Sajna SA, Sandhu G. Joseph, Arjun Chand CP, Sreejith Satheesan, Arun Pratap, Nishanth Kumar S, Jerin Paul, Vipin Nair V, Vijaya R, Hareendran Nair J

**Affiliations:** a HCEMM-SU Cardiovascular Comorbidities Research Group, Department of Pharmacology and Pharmacotherapy, Semmelweis University, 1089, Budapest, Hungary; b Department of R&D, Pankajakasthuri Herbal Research Foundation, Pankajakasthuri Ayurveda Medical College Campus, Trivandrum, India; c Department of Kayachikitsa, Pankajakasthuri Ayurveda Medical College & PG Centre, Killy, Kattakada, Thiruvananthapuram, Kerala, India; d Pankajakasthuri Herbals India Pvt. Ltd., Poovachal, Trivandrum, India; e Department of Rasashastra & Bhaishajya Kalpana, Pankajakasthuri Ayurveda Medical College & P.G. Centre, Killy, Kattakada, Thiruvananthapuram, Kerala, India; f Department of Dravyagunavijnanam, Pankajakasthuri Ayurveda Medical College & P.G. Centre, Killy, Kattakada, Thiruvananthapuram, Kerala, India; g Department of Shalyatantra, Pankajakasthuri Ayurveda Medical College & PG Centre, Killy, Kattakada, Thiruvananthapuram, Kerala, India; h Department of Statistics, Vimala College (Autonomous), Thrissur, Kerala, 680009, India; i Neyyar Medicity, Killy, Kattakada, Thiruvananthapuram, Kerala, India

**Keywords:** DiabetEaze powder, Nutritional supplement, Malnutrition, Microminerals, Vitamins, Diabetes, Sugar spikes

## Abstract

**Background and aims:**

Recently Diabetes Mellitus (DM) has been associated with heightened susceptibility to malnutrition, suggesting that augmenting nutritional intake stands out as a potent therapeutic strategy for addressing malnutrition in individuals with DM. The aim of this clinical investigation was to evaluate the effect of DiabetEaze powder, a polyherbal nutritional formulation developed by us for nutritional management and glycaemic control, on patients with diabetic and prediabetic conditions.

**Methods:**

A total of 143 type II diabetic (T2D) patients who were managing their diabetic condition through modern medicine, AYUSH medicine, lifestyle modification and 68 pre-diabetic patients, aged between 40 and 65 years, were randomly assigned into six groups: control, modern, AYUSH, lifestyle, prediabetic control and prediabetic trial. The treatment groups were administered 5 g of DiabetEaze powder two times a day after food for 6 months. Microminerals, vitamins, glycaemic parameters, Quality of Life (QoL), hematology, lipid profiles, Renal Function Test (RFT) and Liver Function Test (LFL) parameters, and electrolytes were evaluated at Day 0, Day 90, and Day 180.

**Results:**

Out of 211 enrolled patients, 189 individuals successfully completed the entire 180-day duration of the study, indicating a retention rate of approximately 89.6 %. In our study, we observed a statistically significant elevation in the levels of vitamin D, B2, and B6 across all treatment groups. Besides, the treatment groups displayed a notable increase in zinc and manganese levels compared to the other minerals tested. Notably, the treatment groups demonstrated distinct mineral and vitamin profiles. In terms of metabolic markers, significant reductions in Fasting Blood Sugar (FBS)/Post Prandial Blood Sugar (PPBS) were observed across the modern, AYUSH, and lifestyle groups, while the modern group also showed a marked decrease in glycated haemoglobin (HbA1c) levels. Furthermore, overall QoL among the tested groups was also statistically significant. The consistent maintenance of normal LFT and RFT parameters and electrolyte levels across trial groups throughout the study duration indicates that the supplement does not induce liver toxicity or negatively impact hepatic function.

**Conclusion:**

In conclusion, the nutrients present in the DiabetEaze powder contribute to the effective management of nutritional status in diabetic people and thus effectively reduce sugar spikes by regulating PPBS and HbA1c levels, which is a critical aspect of its role in diabetes management. These properties benefit in managing diabetes-related outcomes and overall quality of life.

**Clinical trial registry of India under registration no:**

CTRI/2021/04/032956 on 20/04/2021.

## 1 Introduction

Type 2 Diabetes (T2D) is an extremely predominant metabolic disorder which affects 9.3 % of 20 to 79-year-old adults worldwide and projections increase every year. T2D emerges as a significant public health challenge of the 21^st^ century. According to recent estimations by the International Diabetes Federation (IDF), the global prevalence of T2D is estimated at 578 million individuals, i.e. 10.2 % of the global population, would be diabetic by the end 2030 [1,2] Recent projections from the IDF suggest that this prevalence could escalate to 10.9 %, equivalent to approximately 700 million individuals, by the end of the 2045 [2,3]. The global incidence of T2D exhibiting a skyrocketing increase and it is accounting for the majority of diabetes cases, ranging from 90 to 95 % [3,4]. India is known as the diabetes capital of the world, with an estimated 77 million people affected by DM. The prevalence of diabetes in India is around 8–10 % of the adult population. A recent study published in The Lancet suggests that approximately 101 million individuals in India, constituting 11.4 % of the country's population, are affected by diabetic conditions [5]. A survey conducted by the Health Ministry, Govt. of India also recorded that 136 million people or 15.3 % of the people could be living with pre-diabetic condition. The incidence of DM in India is experiencing a notable increase, with a concerning trend being the transition of onset age from adulthood to adolescence, as reported by Suvarna et al. [6]. Such a shift may impose significant burdens on both the nation's healthcare system and economy.

Malnutrition, increasingly recognized as a significant comorbidity in DM, is associated with heightened susceptibility to geriatric syndromes, including sarcopenia, which further exacerbates glucose dysregulation and accelerates disease progression [[7], [8], [9]]. The dual burden of malnutrition and DM, particularly in T2D and prediabetes, profoundly impacts metabolic, hormonal, and cellular mechanisms critical for maintaining glucose homeostasis [10]. Undernutrition, marked by inadequate intake or absorption of macronutrients and essential micronutrients, contributes to pancreatic β-cell dysfunction and impaired insulin secretion leads to DM [11]. Deficiencies in micronutrients such as magnesium, chromium, zinc, and vitamin D—key regulators of insulin sensitivity and glucose metabolism—further disrupt glycemic control [[12], [13], [14]]. For example, magnesium acts as a cofactor in enzymatic pathways involved in glucose transport and utilization, while chromium enhances insulin receptor activity, improving peripheral glucose uptake [15]. Deficiencies in these nutrients have been directly linked to increased glycemic variability, oxidative stress, and the progression of diabetes-related complications, such as neuropathy, nephropathy, and cardiovascular dysfunction [16].

In populations with T2D and prediabetes, malnutrition frequently coexists due to a combination of poor dietary habits, socioeconomic challenges, and disease-related factors, including altered appetite, gastrointestinal complications, or medication side effects. The prevalence of malnutrition in diabetic populations varies widely, with undernutrition more common in resource-limited settings and overnutrition prevalent in developed regions [17]. Despite the general association of T2D with overweight or obesity, a significant subset of patients, particularly the elderly, experience malnutrition or are at heightened risk [[17], [18], [19]]. Older adults with DM are particularly vulnerable to micronutrient deficiencies, with studies reporting a prevalence of malnutrition among the elderly ranging from 12.0 % to 77.1 % [[19], [20], [21], [22]]. This elevated risk is even more pronounced among elderly individuals with DM compared to those without [20]. Furthermore, over 20 % of hospitalized older individuals with diabetes suffer from malnutrition, which, if unaddressed, leads to poorer clinical outcomes, impaired prognosis, and prolonged hospital stays [23].

Micronutrient deficiencies, particularly vitamins and microminerals, are rampant among diabetic patients due to numerous interconnected factors related to the pathophysiology of diabetes, lifestyle factors, and the side effects of diabetes medications [12]. Prevalence rates of vitamins vary, with estimates ranging from 58 to 63 % for vitamin B6, 13–55 % for vitamin C, 85–91 % for vitamin D [24]. These deficiencies contribute to heightened oxidative stress, inflammation, and immune dysregulation [25]. Given their significance, nutritional management plays a critical role in diabetes care, addressing altered metabolism and increased nutrient requirements. Diabetic patients require customised dietary approaches to mitigate deficiencies arising from increased urinary excretion, impaired absorption of nutrients, or inadequate dietary intake. In T2D, nutritional deficiencies are linked to the progression from β-cell dysfunction to loss of β-cell mass, subsequently leading to impaired insulin signaling and hyperinsulinemia [25]. Furthermore, micronutrients serve as essential cofactors in regulating cellular mechanisms involved in stress response and reactive oxygen species (ROS) production [12]. Disruption in the insulin signalling pathway and increased ROS production, mainly associated with lipoxygenase activity, can occur due to micronutrient deficiencies. These disruptions are the major underlying factors in the development of diabetic and peripheral neuropathy [26]. Specifically, inadequate levels of micronutrients compromise the body's ability to manage oxidative stress and maintain proper cellular function, leading to the progression of neuropathic complications in diabetic individuals. Hence these deficiencies can worsen most of the diabetic complications and impair overall health. Hence a balanced intake of vitamins and minerals is essential for maintaining optimal glycaemic control. Thus, well-balanced nutritional formula can enhance overall well-being, improve energy levels, and boost immunity, which is pivotal for diabetic patients. Given the unique nutritional needs and increased risk of complications in diabetic patients, there is a pressing need for an effective nutritional formula rich in vitamins and minerals. Such a formula can play a pivotal role in managing diabetes, improving QoF, and reducing the risk of complications.

Nutritional interventions are central to the effective management of T2D and prediabetes, focusing on optimizing glycemic control, reducing the risk of complications, and improving overall health outcomes [27]. Existing strategies highlight dietary modifications such as the incorporation of low-glycemic-index foods, balanced macronutrient distribution, and increased dietary fiber intake, all of which help regulate postprandial glucose levels and enhance insulin sensitivity. Caloric restriction and weight loss, particularly in overweight or obese individuals, have been shown to reverse insulin resistance and delay the progression from prediabetes to T2D [28]. Micronutrient supplementation, particularly with magnesium, chromium, zinc, and vitamin D, is often employed to address deficiencies that impair glucose metabolism and insulin action [12]. Functional foods and nutraceuticals, enriched with bioactive compounds such as polyphenols, flavonoids, and omega-3 fatty acids, have garnered attention for their ability to modulate glycemic responses, mitigate systemic inflammation, and counter oxidative stress—key drivers of diabetes progression [29]. Additionally, emerging therapies leveraging probiotics and prebiotics aim to restore gut microbiota balance, which is increasingly recognized as a modulator of glucose metabolism and insulin resistance [30].

Despite these advancements, current nutritional interventions face challenges, including poor patient adherence, limited accessibility in resource-constrained settings, and an inability to fully address the complex nutritional deficiencies often observed in diabetic populations. In this context, our R&D department has developed Pankajakasthuri DiabetEaze powder, a novel polyherbal formulation, offers a promising alternative by combining traditional knowledge with modern therapeutic principles as a nutritional adjunct for diabetic and prediabetic populations. This formulation incorporates bioactive phytochemicals from scientifically validated herbs, targeting key metabolic pathways to regulate glycemia, support pancreatic β-cell function, and address micronutrient deficiencies common in individuals with T2D and prediabetes. Its potential to enhance insulin sensitivity, combat oxidative stress, and improve overall nutritional status positions it as a holistic and accessible intervention for comprehensive diabetes management.

The formulation includes carefully selected herbs such as *Curcuma longa Linn*, *Emblica officinalis Gaertn*, *Strychnos potatorum Linn*, *Salacia reticulata Linn*, *Carum carvi Linn*, *Mimosa pudica*, *Moringa oleifera*, *Tinospora cordifolia*, *Andrographis paniculata*, *Artocarpus heterophyllus*, *Eleusine coracana*, *Hordeum vulgare*, *Anacardium occidentale*, and *Theobroma cacao*, combined in specific proportions. Many of these ingredients have established antidiabetic properties [[31], [32], [33], [34], [35], [36], [37]] and also function as nutraceuticals [[38], [39], [40], [41]] Furthermore, ingredients like *A. heterophyllus*, *E. coracana*, *T. cacao*, *A. occidentale*, *H. vulgare*, and *M. oleifera* are rich in essential vitamins, minerals, trace elements, and amino acids, making them beneficial for nutritional management in diabetes [[40], [41], [42], [43], [44], [45], [46], [47]]. The herbal components used in the formulation are also abundant in polyphenols, which possess antioxidant and anti-inflammatory properties and have been extensively studied for their therapeutic potential in diabetes. Polyphenols can improve insulin sensitivity, enhance glucose uptake, and regulate blood sugar levels [35,48,49]. These multifaceted benefits underscore the significance of DiabetEaze powder as a comprehensive nutritional and therapeutic intervention for diabetic and prediabetic individuals.

This six-arm randomized, open-label, comparative, multi-center, investigator-initiated clinical trial intended to assess the effects of 5 g of DiabetEaze power, an herbal nutritional powder (taken two times per day) on nutritional and glycemic management in T2D and prediabetic patients. To the best of our knowledge, this is the first clinical trial to investigate the impact of herbal nutritional formulation on nutritional management, glycemic control, and safety in individuals with T2D who were managing their condition through various approaches, including modern medicines, AYUSH medicine and lifestyle modifications.

## 2 Materials and methods

### 2.1 Formulation of DiabetEaze powder - the study drug

DiabetEaze powder was manufactured and provided by the production line of Pankajakasthuri Herbals India Pvt. Ltd., Trivandrum, Kerala, India. The ingredients used for the formulation of DiabetEaze powder are shown in Table 1 and the micro/macronutrients and caloric values of the DiabetEaze powder are shown in Table 2. The herbal ingredients used for the preparation of DiabetEaze powder were identified and authorised by Dr. R. Vijaya, Professor, Department of Materia Medica & Pharmacology, Pankajakasthuri Ayurveda Medical College & PG Centre, Trivandrum, Kerala, India. A voucher specimen of the plant was preserved for reference in the Central Herbarium of the Pankajakasthuri Ayurveda Medical College & PG Centre.

**Table 1:** Ingredients used for the formulation of DiabetEaze powder.

S.No.	Ingredients	Part used	Quantity
1	*Curcuma longa*	Rhizome	500 mg
2	*Embilica officinalis*	Fruit	500 mg
3	*Strychnos potatorum*	Seed	500 mg
4	*Salacia reticulata*	Root	500 mg
5	*Carum carvi*	Seeds	500 mg
6	*Mimosa pudica*	Whole plant	300 mg
7	*Moringa oleifera*	Seeds and leaf	300 mg
8	*Tinospora cordifolia*	Stem	300 mg
9	*Andrographis paniculata*	Whole plant	300 mg
10	*Artocarpus heterophyllus*	Whole fruit	300 mg
11	*Eleusine coracana*	Seeds	300 mg
12	*Hordeum vulgare*	Fruit	300 mg
13	*Anacardium occidentale*	Nut	200 mg
14	*Theobroma cacao*	Seeds	200 mg

**Table 2:** Nutritional level of DiabetEaze powder.

Parameters	Level
**Nutritional Facts**	
Carbohydrate	71.52 g/100 g
Energy value	389.36 Kcal
Total fat	6.72 g/100 g
Total protein	10.70 g/100 g
Total sugar as Sucrose	0.00
Crude fibre	12.95 g/100 g
Moisture	5.91 g/100 g
**Vitamins**	
Vitamin B1	17 mcg
Vitamin B2	9 mcg
Vitamin B3	4.20 mcg
Vitamin B5	0.17 mcg
Vitamin B6	50 mcg
Vitamin B9	0.10 mcg
Vitamin B12	38.7 mcg/5 g
Vitamin C	48.43 mg/100 g
Vitamin A	250IU/100 g
Vitamin D	1.1mcg
**Minerals**	
Phosphorous	85.7 mg/100 g
Chromium	6.78 mcg
Molybdenum	5.1mcg
Calcium	229.8 mg/100 g
Zinc (as Zn)	3.78 mg/100 g
Magnesium	158.7 mg/100 g
Manganese	3.19 mg/100 g
Copper	900 mcg/100 g
Selenium	18 mcg/100 g
Iron	8 mg/100 g
Sodium	339.39 mg/100 g
Potassium	648.75 mg/100 g

The formulation of DiabetEaze powder was developed following a structured process guided by Ayurvedic principles and supported by modern pharmacological evidence. The total amount of powder per dose is 5 g, with all ingredients serving as active components, ensuring maximum therapeutic efficacy without the inclusion of additives or excipients. The selection of ingredients was based on strong evidence drawn from classical Ayurvedic texts [[50], [51], [52], [53]], which outline the properties and therapeutic applications of herbs. This traditional knowledge was further corroborated by modern scientific publications that highlight the phytochemical composition, hypoglycemic potential, and nutritional benefits of the selected herbs [[31], [32], [33], [34], [35], [36], [37],[40], [41], [42], [43], [44], [45], [46], [47]] The collected plant materials used for the formulation were individually washed thoroughly and dried in sun shade at room temperature. After that, dried plant materials were separately pulverized to make powder and passed through sieve no. 40. All the pulverized plant materials, as per formula were mixed well by using a double-cone blender. Post-blending, the resultant powder was subjected to drying in a hot air oven at 40 °C for a duration of 1 h and then sealed in an airtight container for further investigations.

### 2.2 Trial organization

The sponsor played no role in the study's design, data collection, analysis, interpretation, or manuscript preparation. An independent data and safety monitoring board oversaw the trial's conduct and safety to safeguard patient well-being.

### 2.3 Study settings

The present study was a six-arm randomized, open-label, comparative, multi-center, investigator-initiated clinical trial to assess the efficacy and safety of Pankajakasthuri DiabetEaze powder for nutritional management in adults diagnosed with T2D and prediabetic conditions. The study sites are Pankajakasthuri Ayurvedic Medical College and PG Centre, Kattakada, Trivandrum, Kerala, India and Neyyar Medicity, Kattakada, Trivandrum, Kerala, India. The design, execution, and coordination of the study were overseen by the Clinical Research Department of Pankajakasthuri Herbal Research Foundation, which was responsible for appointing study officials, trial professionals, data management team and subcommittee members. All subjects were given ample details of the study, including any benefits and risks of participating in the trial. Participants are provided with detailed informed consent form (ICF) of the study, including its objectives, duration, and procedures involved. Participants are given ample time (24 h) to review the ICF thoroughly. Further they were encouraged to ask questions and seek clarification regarding any aspects of the investigation which they do not understand. Finally written informed consent was obtained from all the subjects. The informed consent process was reviewed and approved by Independent Ethics Committee (IEC) of Pankajakasthuri Herbal Research Foundation to ensure that it meets ethical standards and protects the rights and welfare of participants. The study obtained clearance and approval from the IEC of the Pankajakasthuri Herbal Research Foundation, which approved the protocol (Protocol No: PHRF/IEC/001/2021). The study was registered in the Clinical Trial Registry of India under Registration No. CTRI/2021/04/032956. The study was conducted in accordance with the principles of the Declaration of Helsinki and Good Clinical Practice.

### 2.4 Recruitment of subjects

The recruitment of subjects started in July 2021 and the final recruitment was in March 2023. The subjects were recruited via advertisements disseminated through print, audio-visual, and social media platforms. All documentation and advertising materials obtained ethical committee approval and were archived for future reference. Prior to dissemination, all promotional materials and documentation underwent rigorous scrutiny and approval by the IEC of Pankajakasthuri Herbal Research Foundation. Subsequently, these sanctioned materials were systematically archived to ensure traceability and facilitate future reference within the research framework. Patients meeting the study criteria are asked to provide written informed consent, which comprehensively outlines the nature, objectives, potential benefits, and risks of the study. Moreover, the consent form specifies the duration of follow-up, available supportive care, identifies the principal investigators overseeing the protocol, and emphasizes the patient's rights to accept or decline treatment and to withdraw from the study at any point.

### 2.5 Trial design

The present study was a six-arm randomized, open-label, comparative, multi-center, investigator-initiated clinical trial to evaluate the safety and efficacy of Pankajakasthuri DiabetEaze Power for nutritional management in adults diagnosed with T2D and prediabetic conditions.

### 2.6 Inclusion criteria

Subjects were included if they were willing to sign a consent form; men and women aged 40 years or older and not older than 65 years; subjects with clinical evidence of T2D; subjects of pre-diabetes with an HbA1c range between 5.7 % and 6.4 % and/or OGTT range between 140 mg/dl and 199 mg/dl.

### 2.7 Exclusion criteria

Subjects were excluded if they had the presence of conditions like medical, psychological, social or alcohol abuse that would interfere with their participation in the trial; Subjects with a known allergic to any of the ingredients in the drug product; Pregnant or lactating women; subjects with Type 1 DM; subjects undergoing insulin therapy; subjects with fluctuating blood sugar levels; other conditions like chronic kidney disease (CKD), malignancies, etc. Post-surgical cases, mainly gastrointestinal diseases; Patients under steroid intake.

### 2.8 Randomization and allocation

**Fig. 1:**
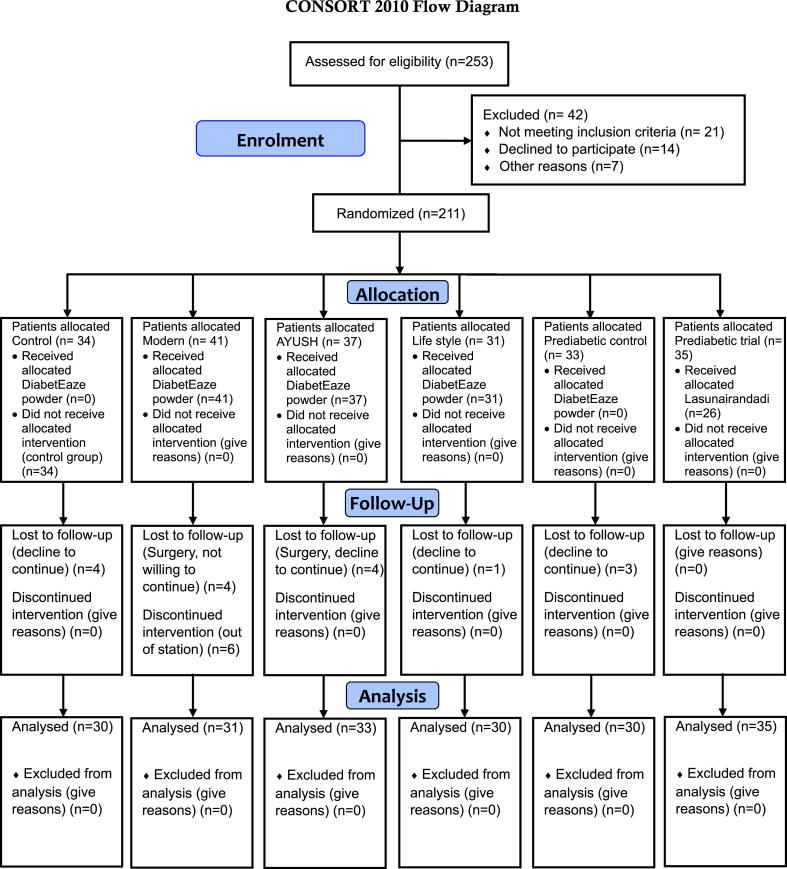
Trial flow chart of the study.

In total, 253 patients were screened in the diabetic outpatient department (OPD), and after exclusion criteria, 211 patients were enrolled in the study. The detailed enrollment procedures were provided in Fig. 1. The randomization process for this study was designed to ensure systematic and unbiased allocation of the 211 enrolled participants into six distinct groups: control, AYUSH, modern medicine, lifestyle intervention, prediabetic control, and prediabetic group (Fig. 1). Initially, all enrolled patients underwent a thorough evaluation, including detailed assessments of their medical history, physical examination, vital sign measurements, electrocardiogram, and laboratory parameter testing including current management strategies, and glycemic status (T2D or prediabetes). These evaluations were documented in individual files, which were then reviewed by a panel of expert doctors. The panel categorized each participant into an appropriate group based on their current management approach, such as the use of modern medicine, AYUSH treatments, or lifestyle interventions. This categorization ensured that participants were grouped according to their ongoing treatment modalities to maintain the integrity of comparisons within the study. Once participants were assigned to their preliminary categories, randomization within each group was carried out using computer-generated random sequences. This process helped to evenly distribute participants and mitigate potential biases while maintaining the treatment modality they were already following. For instance, participants in the modern medicine group were individuals already managing diabetes with modern pharmacological treatments, while those in the AYUSH group were managing their condition using Ayurvedic or other traditional approaches. Similarly, participants in the lifestyle intervention group were undergoing structured dietary and exercise modifications. Prediabetic participants without any active medical treatment were assigned to either the prediabetic control group or the prediabetic treatment group. Importantly, patients were not shifted between groups after enrollment to preserve the continuity of their existing management strategies. This careful grouping and subsequent randomization ensured that the study captured the real-world effectiveness of various interventions while minimizing confounding factors. The process also enabled a robust comparison of the outcomes associated with DiabetEaze powder alongside other treatment modalities, thus enhancing the reliability and clinical relevance of the findings.

By incorporating subjects undergoing diverse management strategies, encompassing modern pharmaceuticals, AYUSH therapies, and lifestyle interventions for diabetes management, the study enables a comparative analysis of the efficacy of the nutritional supplement across varied treatment methods.

• Group 1: This group serves as the control group for the modern medicine, AYUSH and lifestyle intervention group. This group received no DiabetEaze powder and served as a baseline for comparison.
• Group 2: This group was administered DiabetEaze powder as a nutritional supplement as an adjunct therapy for patients managing diabetes through modern medicines.
• Group 3: This group was administered DiabetEaze powder as a nutritional supplement as an adjunct therapy for patients managing diabetes through AYUSH medicines.
• Group 4: This group was administered by DiabetEaze powder as a nutritional supplement as an adjunct therapy for patients managing diabetes through lifestyle interventions such as dietary modifications, regular physical activity, weight management, and stress reduction techniques.
• Group 5: Control group for the prediabetic group. This group received no DiabetEaze powder and served as a baseline for comparison.
• Group 6: This group was administered DiabetEaze powder as a nutritional supplement in the prediabetic group.

These six arms were selected to compare the impact of DiabetEaze powder on nutritional management in patients who are managing their diabetic conditions through various approaches.

### 2.9 Study protocol and intervention

The 211 enrolled subjects were randomly allocated to trial groups and control groups and followed for 180 days. The following data were recorded as baseline assessments: demographics (age, gender, height, and weight), diabetes and comorbidities, nutritional status, and QoF. Laboratory measurements (blood markers including vitamins and minerals) were also obtained according to standard practice at each participating site. The detailed methodology used for analysis vitamins, minerals, glycemic index, hematology and other serum biochemistry parameters is provided in the Supplementary Table 1. Systolic blood pressure (SBP) and diastolic blood pressure (DBP) were reassessed after 15 min of seated rest using a conventional sphygmomanometer (Diamond), with the average of the two measurements considered. Three visits were conducted by the patients: a baseline visit, an intermediate visit on the 90^th^ day and a final visit at 180 days.

The enrolled subjects in the treatment groups were advised to administer DiabetEaze powder orally at a dose of 5 g along with a sufficient quantity of drinking water twice daily, 1 h after food. No intervention was given in the control group. The subjects in the trial groups, except the pre-diabetic and lifestyle groups, were instructed to continue their regular antidiabetic medications and life style modification. Throughout the trial, patients were instructed not to alter their oral hypoglycaemic medications unless specifically advised by their personal physician. Additionally, they were advised against modifying their regular dietary or physical activity routines or taking any supplements. Regular telephone interviews were conducted every 15 days to monitor subjects' progress. All treatments were administered over a six-month period. Detailed dietary instructions were individually provided to each subject by a nutritionist. To ensure that all patients adhered to the recommended use of DiabetEaze powder during the study period, a multi-faceted approach was employed. At the initiation of the trial, participants received detailed counseling from the study personnel, where clear verbal and written instructions were provided regarding the preparation, dosage, and timing of consumption of DiabetEaze powder. Each participant was given a diary to document their daily usage, noting the time of administration, quantity consumed, and any deviations from the prescribed regimen. These diaries were meticulously reviewed during scheduled follow-up visits, where participants were further counselled on adherence and the importance of compliance to the study protocol. To monitor adherence, pre-measured of DiabetEaze powder were distributed at each follow-up, and participants were required to return unused bottles at subsequent visits. The returned bottles were cross-verified against the expected consumption to assess compliance. In addition, periodic phone calls were made to participants between visits to remind them of the importance of regular consumption and to address any challenges they faced. These measures ensured consistent usage of DiabetEaze powder, minimizing variability in intervention adherence and enhancing the reliability of the study outcomes. Additionally, subjects were encouraged to promptly report any adverse effects experienced.

### 2.10 Outcome assessments

#### 2.10.1 Anthropometric

Anthropometric measurements, encompassing body weight (kg) and height (cm), were obtained at baseline, on the 90th day, and at the conclusion of the study, adhering to standard protocols. Body Mass Index (BMI) was derived by dividing weight (kg) by height squared (mˆ2). Blood pressure was assessed twice at each time point (baseline and study end) with a 5-min interval between readings, utilizing an electronic OMRON machine (Omron HEM 7120, Tokyo, Japan). Subjects were comfortably seated with straight backs and feet flat on the floor, and the average of the two readings was recorded.

#### 2.10.2 Primary outcome

##### 2.10.2.1 Nutritional assessment

The major primary outcomes of the current study include the assessment of the nutritional status of the subjects, which comprises the level of 4 vitamins (Vitamin B6, B2, B12 & D3) and 7 major microminerals (magnesium, copper, calcium, phosphorus, manganese, zinc & selenium) in each treatment group at baseline (0^th^ day), 90^th^ day and end of the study (180^th^ day). The nutritional changes, including alterations in vitamin and mineral levels, often require 8–12 weeks duration to manifest due to the time needed for metabolic adjustments and physiological assimilation.

##### 2.10.2.2 Glycemic parameters

Blood glucose levels, including FBS and PPBS, were measured using the hexokinase method, while HbA1c was quantified through the turbidimetric inhibition immunoassay (TINIA) technique. These assessments were performed at baseline (Day 0), Day 90, and Day 180. HbA1c serves as a key indicator of long-term glycemic control, reflecting the average plasma glucose levels over the preceding 8–12 weeks due to its correlation with the lifespan of red blood cells. This timeframe aligns with established scientific evidence that significant changes in HbA1c levels are typically observed over 90-day intervals, justifying the chosen evaluation schedule [54].

An Oral Glucose Tolerance Test (OGTT) was also conducted at these time points, requiring participants to consume a 75 g glucose solution dissolved in 350 mL of filtered tap water within 15 min. Additionally, QoL assessments were carried out using the **Quality of Life Instrument for Indian Diabetes Patients (QOLID),** which comprises 34 items across eight domains as validated by John et al. [55].

#### 2.10.3 Assessment of safety parameters

The safety evaluation encompasses various parameters: (1) measurement of vital signs, including temperature, blood pressure, respiration, and heart rate; (2) routine blood tests including Haemoglobin, Packed Cell Volume, Red Cell Count, Platelets, Reticulocyte Count, total leucocyte count, Neutrophils, Lymphocytes, Eosinophils, and erythrocyte sedimentation rate (ESR); (3) LFT such as Alanine aminotransferase (ALT), aspartate aminotransferase (AST), alkaline phosphatase (ALP), total protein, serum albumin, serum globulin, and albumin creatinine ratio; (4) RFT including urea, serum creatinine, serum uric acid, and total bilirubin; (5) lipid profile encompassing total cholesterol (TC), triglyceride (TG), high-density lipoprotein (HDL), and low-density lipoprotein (LDL); (6) serum electrolytes (sodium, potassium, bicarbonates, and chlorides); and (7) documentation of adverse events at any time. Safety parameters were assessed at baseline (0^th^ day) and end of the study (180^th^ day).

### 2.11 Handling of withdrawal and data management

Participants have the option to discontinue their participation in the study at any juncture and for any reason. In the event of withdrawal, clinicians inquired whether patients were willing to complete assessments as per the study schedule and documented the final day of formulation administration. Instances of patients losing follow-up or withdrawing from the study were meticulously documented and reported. Data gathered during this trial are documented on case report forms, with information being entered using the double-entry method subsequent to each visit at the respective centers.

### 2.12 Adverse events

Adverse events (AEs) are defined as negative or unexpected clinical manifestations of treatment. Patients were asked to notify the investigators of any abnormal reactions they experienced at any time during the trial. In addition, investigators collected information about abnormal reactions on a monthly basis. CRFs were used to record all details of related and unexpected AEs, including the time of occurrence, degree and duration of the AEs, suspected causes, and effective measures and outcomes. Any AEs, such as subjective discomfort and laboratory abnormalities, were treated seriously. Careful analysis and immediate action are taken to ensure the subjects' safety until the adverse events are resolved. In addition, a data safety monitoring board will oversee the trial.

### 2.13 Sample size calculation

The sample size was initially calculated as 31 subjects in each group using the G∗Power software based on the F test. For this calculation, α level of 5 % and a power of 90 % were utilized, with an effect size of 0.25. However, to ensure the adequacy of the sample size and account for potential participant dropout, particularly over the three-month follow-up period, the final sample size for each group was increased to 36, considering a dropout rate of 15 %. Before random allocation to the parallel groups, all participants underwent RBS tests in the laboratory. An endocrinologist evaluated the laboratory tests to identify eligible cases.

### 2.14 Statistical analysis

All data are presented as mean ± standard deviation (SD). Comparative analyses of vitamin and mineral levels, as well as glycemic indices across treatment groups, were performed using independent sample t-tests, applying a day-wise and group-wise approach. For non-normally distributed demographic and baseline parameters, the Kruskal-Wallis test was utilized to identify statistically significant differences among groups. The impact of the intervention on quality of life (QoL) was assessed using a Linear Mixed Model approach, accounting for both fixed effects (such as treatment groups) and random effects (individual variability over time), providing a robust analysis of longitudinal QoL data. Significance was defined as a p-value less than 0.05 for all statistical tests. The data analysis was performed using the R statistical software with the JAMOVI interface, ensuring precision and reproducibility of results.

## 3 Results

As shown in Fig. 1, 253 subjects were screened for eligibility (diabetic & prediabetic). Out of these, 42 patients were excluded (not meeting inclusion criteria [*n* = 21], declined to participate [*n* = 14] and for other reasons [*n* = 7]), and finally, 211 diabetic and prediabetic subjects were randomized to the control and treatment groups in the trial (Fig. 1). The justifications for exclusion from the trial are also delineated in Fig. 1. The 211 enrolled subjects were randomly assigned to the six groups. Out of 211 enrolled subjects, 22 dropped out of the study due to various reasons, including having undergone surgery, being out of station, etc. The number of dropouts from each group was also given in Fig. 1. Dropouts were not due to intolerance or side effects, but purely due to personal reasons. Totally, 189 patients completed the 180 days of the study (30 subjects in the control group, 31 subjects in the modern group, 33 subjects in the lifestyle group, 30 subjects in the AYUSH group, 30 subjects in the prediabetic control group & 35 subjects in the prediabetic trial group).

Table 3 summarizes the baseline demographic characteristics of subjects across the six groups, including mean age, gender distribution (male and female numbers), duration of diabetes, body weight, and BMI.

**Table 3:** Baseline parameters of the subjects in the various groups enrolled in the study.

Characteristics	Control (n = 33)	Modern (n = 41)	AYUSH (n = 34)	Lifestyle (n = 32)	Prediabetic Control (n = 33)	Prediabetic (n = 35)	P- value	
Gender Male Female	M - 13 F - 20	M - 17 F - 24	M - 17 F - 17	M - 16 F - 16	M - 10 F - 23	M - 5 F - 30	- -	
Mean age (years)	51.96 ± 8.10	55.16 ± 6.10	51.8 ± 7.63	51.45 ± 7.02	46.9 ± 7.26	47.62 ± 7.24	P ≤ 0.001	
Mean weight (kg)	66.99 ± 9.86	67.27 ± 14.71	71.13 ± 14.36	69.40 ± 9.67	68.16 ± 7.54	63.80 ± 8.73	P ≤ 0.001	
Mean height (cm)	159.97 ± 10.87	160.62 ± 8.76	160.78 ± 9.60	161.60 ± 6.76	158.95 ± 8.17	158.58 ± 5.83	P = 0.741	
Mean body mass index	26.28 ± 3.88	26.01 ± 4.97	27.57 ± 5.33	26.59 ± 3.42	27.11 ± 3.57	23.36 ± 3.30	P = 0.098	
SBP (mmHg)	129.37 ± 11.96	128.19 ± 16.38	131.75 ± 16.45	127.16 ± 10.75	127.26 ± 9.71	123.25 ± 9.83	P = 0.215	
DBP (mmHg)	83.44 ± 8.13	82.12 ± 10.78	86.18 ± 8.50	82.06 ± 8.77	79.66 ± 6.14	78.57 ± 8.09	P = 0.008	
Pulse rate	73.20 ± 7.85	73.90 ± 6.97	75.69 ± 8.37	75.07 ± 6.90	75.26 ± 5.49	73.54 ± 7.23	P = 0.444	
**Clinical presentation**								
Time since diabetes diagnosis (years)	3 Years	5 Years	3 Years	½ Years	NA	NA	-	
Other medications (%)	30.30 %	29.26 %	23.52 %	0	15.15 %	11.42 %	-	
HbA1c (%)	8.6 ± 2.2	8.8 ± 2	9.5 ± 2	8 ± 2	6 ± 0.21	5.98 ± 0.16	P ≤ 0.001	
FBS	190 ± 73.5	193.8 ± 50.3	213.2 ± 68.3	174 ± 69.1	107.9 ± 10.7	107 ± 9.1	P ≤ 0.001	
PPBS	252.13 ± 109.13	221.2 ± 129.8	267.95 ± 113.2	219.4 ± 88.37	117.7 ± 22.3	106.2 ± 15	P ≤ 0.001	
**Comorbidities**								
Hypertension (Nos.)	7	11	13	3	2	2	-	
Hyperlipidaemia (Nos.)	4	5	3	3	-	-	-	
Dyslipidemia (Nos.)	9	9	16	17	16	17	-	
Cardiovascular disease (Nos.)	0	1	0	0	1	0	-	
Renal disease (Nos.)	0	0	0	0	0	0	-	
Intake of any dietary supplements (Nos.)	0	0	0	0	0	0	-	
**Vitamin and Mineral content**								
Vitamin D3 (ng/ml)	18.78 ± 5.04	18.75 ± 6.89	19.81 ± 6.29	18.83 ± 4.49	17.21 ± 4.75	15.93 ± 3.86	P = 0.010	
Vitamin B12 (pg/ml)	863.3 ± 345.5	594.2 ± 397	737.5 ± 389.7	718.1 ± 272.3	493.82 ± 144.76	562.67 ± 218.91	P ≤ 0.001	
Vitamin B2 (Ug/ml)	16.8 ± 10.7	25.1 ± 12.1	15.9 ± 07.5	11.6 ± 05.7	18.6 ± 12.5	12.0 ± 06.1	P ≤ 0.001	
Vitamin B6 (ng/ml)	23.7 ± 13.4	18.6 ± 9.9	19.6 ± 7.1	22.6 ± 8.3	20.3 ± 13.3	13.9 ± 9	P ≤ 0.001	
Calcium (mg/dl)	9.6 ± 0.4	9.5 ± 0.4	9.4 ± 0.5	9.4 ± 0.4	9.25 ± 0.34	9.53 ± 0.35	P = 0.027	
Magnesium (mg/dl)	2.1 ± 0.3	2 ± 0.2	2 ± 0.3	2 ± 0.2	2.02 ± 0.17	2.1 ± 0.16	P = 0.864	
Copper (μg/dl)	121.7 ± 33.2	109.8 ± 27.2	107.8 ± 36.8	128.7 ± 34.6	133.1 ± 22.6	119.5 ± 25.6	P = 0.008	
Manganese (μg/L)	5.9 ± 1.1	5.4 ± 0.7	6.2 ± 1.2	7.5 ± 0.9	8.3 ± 1.9	9.5 ± 2.8	P ≤ 0.001	
Zinc (μg/dl)	73.05 ± 30.11	69.58 ± 33.99	65.47 ± 38.59	73.05 ± 30.11	76.14 ± 16.14	86.29 ± 13.36	P ≤ 0.001	
Phosphorus (mg/dl)	3.9 ± 0.4	4 ± 0.6	3.7 ± 0.4	3.7 ± 0.5	3.84 ± 0.35	3.77 ± 0.43	P = 0.098	
Selenium (μg/L)	214.1 ± 86.7	177.5 ± 66.7	182.2 ± 80.5	154.9 ± 54.6	233.8 ± 73.3	243.3 ± 70.5	P ≤ 0.001	
**Serum Biochemistry**								
Total cholesterol (mg/dl)	187.9 ± 39.55	180.77 ± 34.47	207.73 ± 49.2	203.36 ± 34.53	204.53 ± 34.37	199.94 ± 297	P = 0.059	
HDL (mg/dl)	48.57 ± 11.66	44.19 ± 11.87	47.36 ± 11.60	46.90 ± 8.90	45.63 ± 10.95	46.29 ± 7.96	P = 0.681	
TG (mg/dl)	136.06 ± 69.83	149.19 ± 100.7	150.49 ± 86.23	125.42 ± 35.65	110.3 ± 53.64	102.49 ± 39.49	P = 0.011	
LDL (mg/dl)	112.07 ± 33.77	119.35 ± 58.34	129.94 ± 37.0	128.23 ± 32.1	146.87 ± 68.50	125.77 ± 24.2	P = 0.008	
ALT (IU/l)	34.27 ± 20.85	33.16 ± 17.85	37.55 ± 27.50	25.52 ± 15.28	19.2 ± 11.98	18.66 ± 6.43	P ≤ 0.001	
AST (IU/l)	22.67 ± 10.48	23.68 ± 11.19	26.64 ± 21.02	20.65 ± 6.32	19.13 ± 5.66	18.46 ± 5.1	P = 0.206	
Serum creatinine (mg/dl)	0.74 ± 0.23	0.81 ± 0.65	0.8 ± 0.21	0.79 ± 0.17	0.74 ± 0.20	0.66 ± 0.17	P = 0.028	
Urea (mg/dl)	23.9 ± 7.04	20.97 ± 6.63	23.36 ± 8.05	20.97 ± 5.67	19.97 ± 4.97	19.49 ± 4.77	P = 0.068	
Bilirubin (mg/dl)	0.53 ± 0.26	1.24 ± 3.32	0.63 ± 0.35	0.66 ± 0.37	0.52 ± 0.26	0.57 ± 0.27	P = 0.534	

SBP - Systolic Blood Pressure, DBP- Diastolic Blood Pressure, HbA1c - glycated haemoglobin, FBS- Fasting Blood Sugar, PPBS- Postprandial Blood Sugar, HDL-High-Density Lipoprotein, TG- Triglyceride, LDL- Low-Density Lipoprotein, ALT- Alanine Transaminase, ALP-Alkaline Phosphatase.

### 3.1 Nutritional outcome by the administration of DiabetEaze powder

#### 3.1.1 Impact of DiabetEaze powder on vitamins level

##### 3.1.1.1 Vitamins D3

**Fig. 2:**
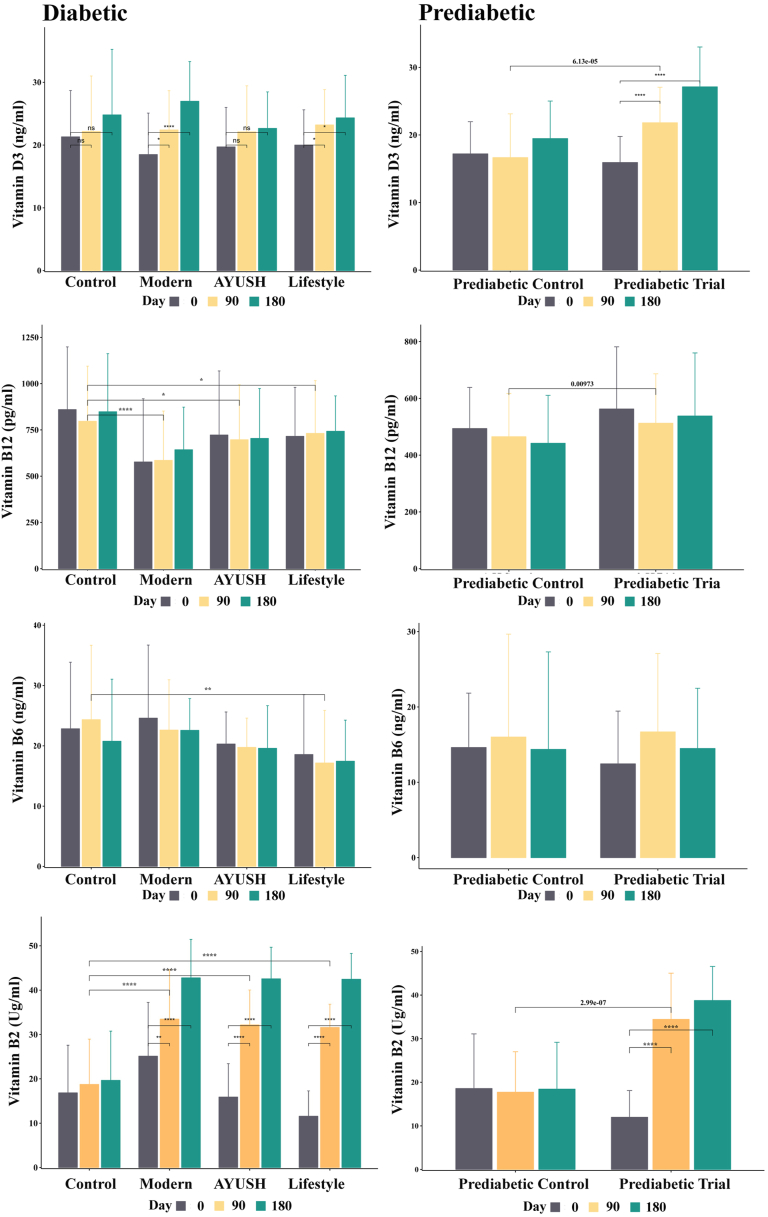
Impact of DiabetEaze powder on the vitamin level in the treatment groups. Error bars represent SEM.

The level of vitamin D3 recorded significant changes over the day-wise intervals across all treatment groups except control (Table 4 & Fig. 2). In day-wise intervals, the lifestyle group recorded significant enhancements in the vitamin D3 level when compared to other treatment groups. Similarly, the prediabetic treatment group also recorded a significant enhancement in the vitamin D3 level in a group-wise comparison. Furthermore, significant differences in the vitamin D3 level were noted between the control vs. modern group (P < 0.05) and the control vs. AYUSH group (P < 0.05) (Table 5). In addition to this, our findings unequivocally demonstrate that the administration of DiabetEaze powder led to a significant elevation in vitamin D3 levels within the treatment groups. Furthermore, significant differences in vitamin D3 levels between the prediabetic control and trial groups (P < 0.05) (Table 4, Table 5 & Fig. 2).

**Table 4:** Day-wise comparative analysis of vitamins levels across treatment groups following administration of DiabetEaze powder.

Variables	Groups	Day 0 Mean ± SD	Day 180 Mean ± SD	Day 90 Mean ± SD	Significant difference between Days		
					Day 0 Vs 90	Day 0 Vs 180	Day 90 Vs 180
Vitamin B2 (Ug/ml)	Control	16.8 ± 10.7	18.8 ± 10.2	19.7 ± 11.1	P = 0.392	P = 0.324	P = 0.726
	Modern	25.1 ± 12.1	33.5 ± 11.1	42.8 ± 08.7	P = 0.009	P ≤ 0.001	P ≤ 0.001
	AYUSH	15.9 ± 07.5	32.2 ± 07.9	42.6 ± 07.1	P ≤ 0.001	P ≤ 0.001	P ≤ 0.001
	Lifestyle	11.6 ± 05.7	31.6 ± 05.2	42.5 ± 05.8	P ≤ 0.001	P ≤ 0.001	P ≤ 0.001
	Prediabetic control	18.6 ± 12.5	17.7 ± 09.3	18.4 ± 10.7	P = 0.295	P = 0.373	P = 0.981
	Prediabetic Trial	12.0 ± 06.1	34.4 ± 10.6	38.8 ± 07.8	P ≤ 0.001	P ≤ 0.001	P = 0.130
Vitamin B6 (ng/ml)	Control	23.7 ± 13.4	25.1 ± 13.4	20.3 ± 5.3	P = 0.746	P = 0.211	P = 0.060
	Modern	18.6 ± 9.9	20.9 ± 10.8	22.6 ± 5.3	P = 0.297	P = 0.265	P = 0.975
	AYUSH	19.6 ± 7.1	17.5 ± 6.8	24.5 ± 12.8	P = 0.605	P = 0.621	P = 0.897
	Lifestyle	22.6 ± 8.3	19.8 ± 4.8	17.2 ± 8.7	P = 0.353	P = 0.534	P = 0.839
	Prediabetic control	20.3 ± 13.3	18.8 ± 11.3	18.7 ± 11.5	P = 0.468	P = 0.373	P = 0.981
	Prediabetic Trial	13.9 ± 9	16.2 ± 10.5	24.9 ± 15.1	P ≤ 0.001	P = 0.259	P = 0.021
Vitamin B12 (pg/ml)	Control	863.3 ± 345.5	850.3 ± 319.1	811.6 ± 344.5	P = 0.173	P = 0.737	P = 0.356
	Modern	594.2 ± 397	653.5 ± 271.5	595.8 ± 304.3	P = 0.947	P = 0.174	P = 0.026
	AYUSH	737.5 ± 389.7	704.3 ± 269.4	699.6 ± 304.1	P = 0.448	P = 0.552	P = 0.878
	Lifestyle	718.1 ± 272.3	742.8 ± 191.1	751.5 ± 348.8	P = 0.584	P = 0.567	P = 0.869
	Prediabetic control	493.82 ± 144.76	441.87 ± 168.88	465.17 ± 151.86	P = 0.324	P = 0.106	P = 0.117
	Prediabetic Trial	562.67 ± 218.91	538.07 ± 221.92	512.64 ± 173.82	P = 0.018	P = 0.329	P = 0.254
Vitamin D3 (ng/ml)	Control	18.78 ± 5.04	20.87 ± 7.94	20.07 ± 8.03	P = 0.389	P = 0.198	P = 0.425
	Modern	18.75 ± 6.89	28.61 ± 12.35	23.37 ± 8.38	P ≤ 0.001	P ≤ 0.001	P = 0.025
	AYUSH	19.81 ± 6.29	26.05 ± 6.14	23.46 ± 5.71	P ≤ 0.001	P ≤ 0.001	P = 0.011
	Lifestyle	18.83 ± 4.49	27.7 ± 4.73	23.86 ± 4.75	P ≤ 0.001	P ≤ 0.001	P ≤ 0.001
	Prediabetic control	17.21 ± 4.75	19.47 ± 5.55	16.65 ± 6.47	P = 0.607	P = 0.025	P = 0.024
	Prediabetic Trial	15.93 ± 3.86	27.13 ± 5.89	21.81 ± 5.26	P ≤ 0.001	P ≤ 0.001	P ≤ 0.001

**Table 5:** Group-wise comparative analysis of the effects of DiabetEaze powder on vitamins, minerals, and glycemic indices. P value less than 0.05 were considered as statistically significant. Control (Cont.), Modern (Mod.), AYUSH (Ayu.), Lifestyle (LS), Prediabetic Control (PDC), and Prediabetic Trial (PDT).

Parameters	Cont. Vs Mod.	Cont. Vs Ayu.	Cont. Vs LS	Mod. Vs Ayu	Mod. Vs LS	Ayu. Vs LS	PDC Vs PDT
**Vitamins**							
Vitamin B2	P ≤ 0.001	P ≤ 0.001	P ≤ 0.001	P = 0.019	P ≤ 0.001	P = 0.077	P ≤ 0.001
Vitamin B6	P = 0.798	P = 0.027	P = 0.002	P = 0.003	P ≤ 0.001	P = 0.019	P = 0.358
Vitamin B12	P ≤ 0.001	P = 0.014	P = 0.019	P = 0.031	P ≤ 0.001	P = 0.624	P = 0.022
Vitamin D3	P = 0.007	P = 0.002	P = 0.034	P = 0.677	P = 0.380	P = 0.293	P ≤ 0.001
**Minerals**							
Calcium	P = 0.152	P = 0.395	P = 0.411	P = 0.702	P = 0.366	P = 0.379	P = 0.564
Copper	P = 0.036	P = 0.004	P = 0.066	P = 0.326	P ≤ 0.001	P ≤ 0.001	P = 0.002
Magnesium	P ≤ 0.001	P = 0.105	P = 0.056	P = 0.220	P = 0.234	P = 0.963	P = 0.588
Manganese	P ≤ 0.001	P ≤ 0.001	P ≤ 0.001	P = 0.002	P ≤ 0.001	P = 0.179	P ≤ 0.001
Phosphorus	P = 0.606	P = 0.012	P = 0.003	P = 0.040	P = 0.005	P = 0.331	P = 0.270
Selenium	P = 0.585	P = 0.050	P = 0.214	P ≤ 0.001	P = 0.469	P ≤ 0.001	P = 0.738
Zinc	P = 0.029	P = 0.096	P ≤ 0.001	P = 0.096	P ≤ 0.001	P ≤ 0.001	P = 0.042
**Glycemic index**							
HbA1c	P = 0.328	P = 0.010	P = 0.021	P ≤ 0.001	P = 0.245	P ≤ 0.001	P ≤ 0.001
OGTT (1 h)	P = 0.048	P = 0.208	P ≤ 0.001	P ≤ 0.001	P = 0.055	P ≤ 0.001	P = 0.005
OGTT (2 h)	P = 0.014	P = 0.597	P ≤ 0.001	P = 0.010	P = 0.137	P ≤ 0.001	P ≤ 0.001
FBS	P = 0.056	P = 0.256	P = 0.002	P ≤ 0.001	P = 0.399	P ≤ 0.001	P = 0.002
PPBS	P = 0.175	P = 0.077	P = 0.003	P ≤ 0.001	P = 0.108	P ≤ 0.001	P ≤ 0.001

##### 3.1.1.2 Vitamin B12

The results from our study showed that all treatment groups recorded no significant changes in vitamin B12 levels over day wise intervals (Table 4 & Fig. 2). However, significant differences were recorded between the different treatment groups. Significant differences were observed between the control and modern groups (P < 0.05), the control and AYUSH groups (P < 0.05), the control and lifestyle groups (P < 0.05), the modern and AYUSH groups (P < 0.05), and the modern and lifestyle groups (P < 0.05) (Table 5). Besides, a slight difference in vitamin B12 levels was recorded between the prediabetic control and trial groups (P < 0.05). In conclusion, no significant changes in vitamin B12 levels were recorded within groups over time, but group comparisons recorded significant variations in vitamin B12 levels in the subjects. Despite these differences, all treatment groups-maintained vitamin B12 levels within normal limits throughout the study period (Table 4, Table 5 & Fig. 2).

##### 3.1.1.3 Vitamin B6

The vitamin B6 levels did not change significantly between day wise intervals in all the treatment groups (Table 4 & Fig. 2). However, there were significant differences in the interactions recorded between the various groups tested. Significant differences in vitamin B6 were recorded between the control vs AYUSH groups (P < 0.05), control vs lifestyle group (P < 0.05), modern vs AYUSH group (P < 0.05), the modern vs lifestyle group (P < 0.05), and the AYUSH vs lifestyle group (P < 0.05) (Table 5 & Fig. 2). Furthermore, there were no significant differences in vitamin B6 levels between the prediabetic control and trial groups. Overall, while there were no significant changes within groups over time, interactions between groups resulted in variations in vitamin B6 levels, with all groups remaining within the normal range throughout the investigation period (Table 4, Table 5 & Fig. 2).

##### 3.1.1.4 Vitamin B2

The results from our study showed that Modern, AYUSH and Lifestyle groups recorded significant changes in vitamin B2 levels over day wise intervals (Table 4 & Fig. 2). Moreover, significant differences were recorded between the different testing groups. Significant differences were observed between the control and modern groups (P < 0.05), the control and AYUSH groups (P < 0.05), the control and lifestyle groups (P < 0.05), the modern and AYUSH groups (P < 0.05), and the modern and lifestyle groups (P < 0.05) (Table 5 & Fig. 2). Likewise, a significant variation in vitamin B2 levels was recorded between the prediabetic control and trial groups (P < 0.05). In conclusion, significant changes in vitamin B2 levels were recorded within groups over time, likewise group comparisons also recorded significant variations in vitamin B2 levels in the subject.

#### 3.1.2 Impact of DiabetEaze powder on micromineral contents

##### 3.1.2.1 Zinc

**Fig. 3:**
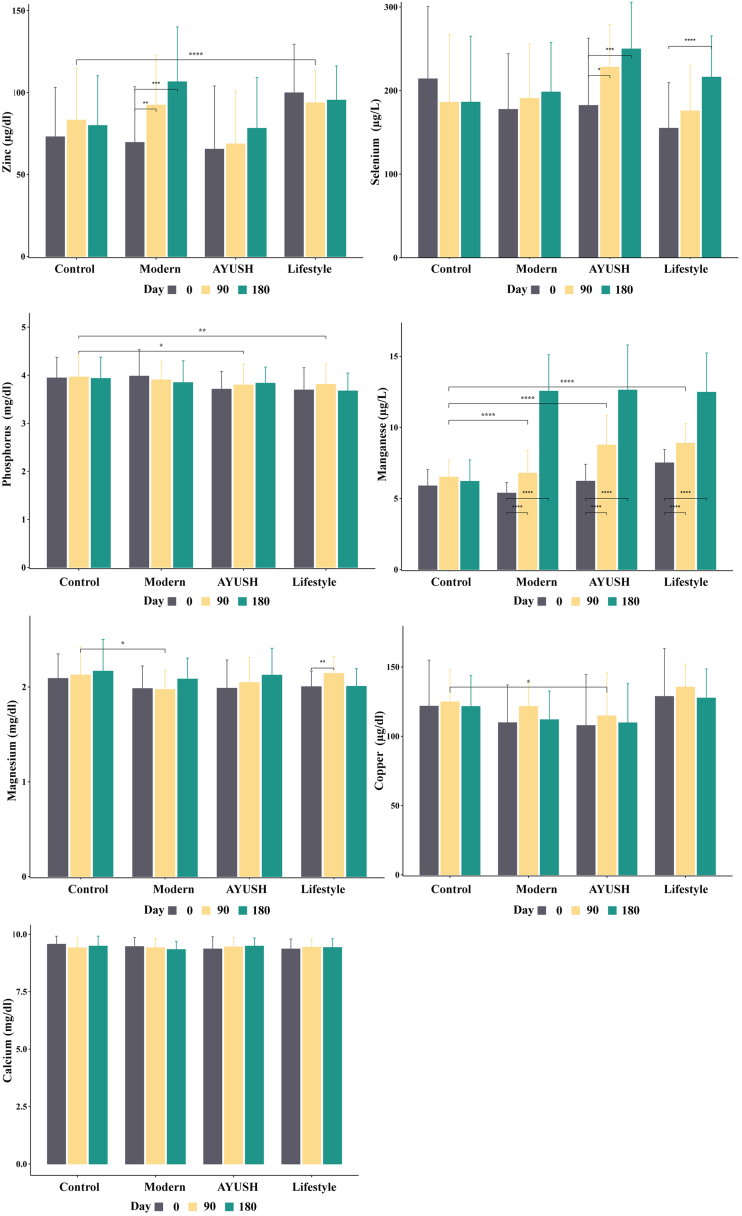
Impact of DiabetEaze powder on the mineral level in the treatment groups (Modern, AYUSH and Lifestyle). Error bars represent SEM.

**Fig. 4:**
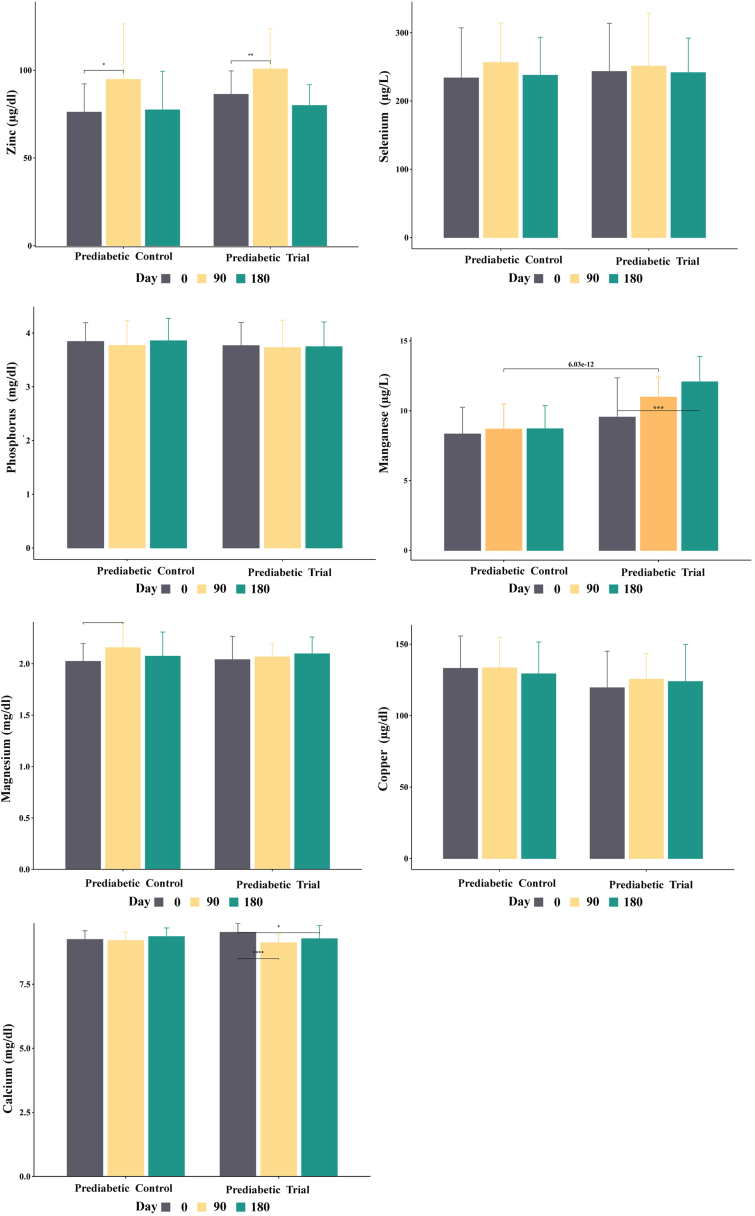
Impact of DiabetEaze powder on the mineral level in the prediabetic group. Error bars represent SEM.

Zinc levels stayed relatively stable in the control, AYUSH, and lifestyle groups when compared with the day wise intervals (Table 6 & Fig. 3). Whereas the modern group recorded significantly higher zinc levels on Day 90 (P < 0.05) and Day 180 (P < 0.05). Similarly, the prediabetic trial group displayed a significant increase in zinc levels from Day 0. In addition to this, significant differences in the zinc level were recorded between the various groups tested. Significant differences were found between the control vs lifestyle groups (P < 0.05), control vs AYUSH (P < 0.05) as well as the control and modern groups (P < 0.05) (Table 5 & Fig. 3). Besides, a slight difference in zinc level was recorded between the prediabetic control and trial groups (Table 5, Table 6 & Fig. 4).

**Table 6:** Day-wise comparative analysis of mineral levels across treatment groups following administration of DiabetEaze powder.

Variables	Group	Day 0 (Mean ± SD	Day 180 (Mean ± SD)	Day 90 (Mean ± SD)	Significant difference between Days		
					Day 0 Vs 90	Day 0 Vs 180	Day 90 Vs 180
Calcium (mg/dl)	Control	9.6 ± 0.4	9.5 ± 0.4	9.4 ± 0.5	P = 0.082	P = 0.362	P = 0.297
	Modern	9.5 ± 0.4	9.3 ± 0.4	9.4 ± 0.4	P = 0.563	P = 0.147	P = 0.335
	AYUSH	9.4 ± 0.5	9.5 ± 0.4	9.5 ± 0.4	P = 0.366	P = 0.233	P = 0.670
	Lifestyle	9.4 ± 0.4	9.2 ± 1.5	9.4 ± 0.4	P = 0.338	P = 0.296	P = 0.326
	Prediabetic control	9.25 ± 0.34	9.36 ± 0.34	9.21 ± 0.33	P = 0.607	P = 0.113	P = 0.019
	Prediabetic Trial	9.53 ± 0.35	9.27 ± 0.52	9.11 ± 0.34	P ≤ 0.001	P = 0.002	P = 0.007
Copper (μg/dl)	Control	121.7 ± 33.2	121.5 ± 22.3	124.8 ± 23.2	P = 0.968	P = 0.434	P = 0.433
	Modern	109.8 ± 27.2	112 ± 20.7	121.6 ± 17	P = 0.543	P = 0.015	P = 0.002
	AYUSH	107.8 ± 36.8	109.7 ± 28.3	114.7 ± 31.2	P = 0.762	P = 0.304	P = 0.074
	Lifestyle	128.7 ± 34.6	127.6 ± 21	135.6 ± 16.5	P = 0.825	P = 0.226	P = 0.002
	Prediabetic control	133.1 ± 22.6	133.5 ± 21.4	129.3 ± 22.2	P = 0.277	P = 0.894	P = 0.219
	Prediabetic Trial	119.5 ± 25.6	125.5 ± 18	123.9 ± 25.9	P = 0.411	P = 0.170	P = 0.691
Magnesium (mg/dl)	Control	2.1 ± 0.3	2.2 ± 0.3	2.1 ± 0.3	P = 0.583	P = 0.340	P = 0.661
	Modern	2 ± 0.2	2.1 ± 0.2	2 ± 0.2	P = 0.853	P = 0.106	P = 0.010
	AYUSH	2 ± 0.3	2.1 ± 0.3	2 ± 0.3	P = 0.258	P = 0.062	P = 0.274
	Lifestyle	2 ± 0.2	2 ± 0.2	2.1 ± 0.2	P = 0.099	P = 0.936	P = 0.002
	Prediabetic control	2.02 ± 0.17	2.04 ± 0.23	2.07 ± 0.23	P = 0.009	P = 0.272	P = 0.067
	Prediabetic Trial	2.1 ± 0.16	2.16 ± 0.25	2.07 ± 0.13	P = 0.467	P = 0.277	P = 0.419
Manganese (μg/L)	Control	5.9 ± 1.1	6.2 ± 1.5	6.5 ± 1.2	P = 0.045	P = 0.371	P = 0.414
	Modern	5.4 ± 0.7	12.5 ± 2.6	6.8 ± 1.6	P ≤ 0.001	P ≤ 0.001	P ≤ 0.001
	AYUSH	6.2 ± 1.2	12.6 ± 3.2	8.8 ± 2.1	P ≤ 0.001	P ≤ 0.001	P ≤ 0.001
	Lifestyle	7.5 ± 0.9	12.5 ± 2.8	8.9 ± 1.4	P ≤ 0.001	P ≤ 0.001	P ≤ 0.001
	Prediabetic control	8.3 ± 1.9	8.7 ± 1.6	8.7 ± 1.8	P = 0.235	P = 0.316	P = 0.960
	Prediabetic Trial	9.5 ± 2.8	12.1 ± 1.8	11 ± 1.4	P = 0.005	P ≤ 0.001	P ≤ 0.001
Phosphorus (mg/dl)	Control	3.9 ± 0.4	3.9 ± 0.4	4 ± 0.4	P = 0.799	P = 0.895	P = 0.760
	Modern	4 ± 0.6	3.8 ± 0.5	3.9 ± 0.4	P = 0.394	P = 0.079	P = 0.455
	AYUSH	3.7 ± 0.4	3.8 ± 0.3	3.8 ± 0.4	P = 0.272	P = 0.095	P = 0.631
	Lifestyle	3.7 ± 0.5	3.7 ± 0.4	3.8 ± 0.4	P = 0.250	P = 0.818	P = 0.109
	Prediabetic control	3.84 ± 0.35	3.86 ± 0.42	3.77 ± 0.46	P = 0.397	P = 0.882	P = 0.241
	Prediabetic Trial	3.77 ± 0.43	3.75 ± 0.46	3.73 ± 0.51	P = 0.647	P = 0.806	P = 0.849
Selenium (μg/L)	Control	214.1 ± 86.7	186.2 ± 78.9	185.9 ± 81.4	P = 0.206	P = 0.254	P = 0.991
	Modern	177.5 ± 66.7	198.3 ± 59.3	190.6 ± 64.9	P = 0.463	P = 0.154	P = 0.637
	AYUSH	182.2 ± 80.5	249.8 ± 56	228 ± 51.4	P = 0.017	P = 0.001	P = 0.157
	Lifestyle	154.9 ± 54.6	216.1 ± 49.4	175.7 ± 54.9	P = 0.195	P ≤ 0.001	P = 0.006
	Prediabetic control	233.8 ± 73.3	237.7 ± 55.6	256.4 ± 57.9	P = 0.078	P = 0.704	P = 0.052
	Prediabetic Trial	243.3 ± 70.5	241.5 ± 50.6	251.1 ± 77.8	P = 0.465	P = 0.856	P = 0.369
Zinc (μg/dl)	Control	73.05 ± 30.11	79.9 ± 30.41	83.19 ± 31.67	P = 0.221	P = 0.297	P = 0.707
	Modern	69.58 ± 33.99	106.58 ± 33.47	92.36 ± 30.43	P ≤ 0.001	P ≤ 0.001	P = 0.009
	AYUSH	65.47 ± 38.59	78.2 ± 31.1	68.7 ± 32.37	P = 0.740	P = 0.168	P = 0.134
	Lifestyle	73.05 ± 30.11	79.9 ± 30.41	83.19 ± 31.67	P = 0.221	P = 0.297	P = 0.707
	Prediabetic control	76.14 ± 16.14	77.43 ± 22.02	94.82 ± 31.63	P = 0.738	P ≤ 0.001	P ≤ 0.001
	Prediabetic Trial	86.29 ± 13.36	79.92 ± 11.9	100.73 ± 22.94	P ≤ 0.001	P = 0.036	P ≤ 0.001

##### 3.1.2.2 Selenium

The selenium levels remained relatively stable in the control and modern treatment groups over the day-wise intervals (Table 6 & Fig. 3). However, the AYUSH group displayed a noteworthy upsurge in selenium levels from Day 0 (182.2 ± 80.5) to Day 90 (249.8 ± 56), while the lifestyle group displayed a significant change from Day 0 (154.9 ± 54.6) to Day 180 (175.7 ± 54.9) with a statistically significant difference between Day 90 and Day 180 (P < 0.05). Significant group interactions were recorded between the modern vs AYUSH groups (P < 0.05) and the AYUSH vs lifestyle groups (P < 0.05) (Table 5 & Fig. 3). Overall, while selenium levels remained stable in the control and modern groups, the AYUSH and lifestyle groups showed significant changes over time. In contrast, there were no significant changes in selenium levels recorded over time within the prediabetic control and prediabetic trial groups (Table 5, Table 6 & Fig. 4). In addition to this, no statistically significant interactions were recorded between the prediabetic control and trial groups.

##### 3.1.2.3 Phosphorus

The phosphorus levels remained relatively stable across all treatment groups when compared over day wise intervals, with no statistically significant variation recorded within individual groups (Table 6 & Fig. 3). However, statistically significant grouping interactions were recorded between the control and AYUSH groups (P < 0.05), control and lifestyle groups (P < 0.05), modern and AYUSH groups (P < 0.05), and modern and lifestyle groups (P < 0.05) (Table 5 & Fig. 3). Moreover, no statistically significant differences in phosphorus levels between day intervals were recorded for prediabetic control and trial groups. In addition to this, the interaction between these two groups did not cause any changes in phosphorus levels (Table 6 & Fig. 4). Although no statistical significance was recorded, it is important to note that all groups-maintained phosphorus levels within normal limits throughout the study period (Table 6).

##### 3.1.2.4 Manganese

The manganese levels displayed statistically significant differences over the day-wise intervals in the modern, AYUSH, and lifestyle groups (Table 6 & Fig. 3). Whereas, the control group did not record any variations in manganese levels. Statistically significant grouping interactions were recorded between the control and modern groups (P < 0.05), control and AYUSH groups (P < 0.05), control and lifestyle groups (P < 0.05), as well as between the modern and AYUSH groups (P < 0.05), and modern and lifestyle groups (P < 0.05) (Table 5 & Fig. 3). Furthermore, significant difference in the manganese level was recorded between the prediabetic control and trial groups (P < 0.05) (Table 5, Table 6 & Fig. 4).

##### 3.1.2.5 Magnesium

The magnesium levels recorded relatively stable in the control and AYUSH groups over the day-wise intervals (Table 6 & Fig. 3). However, significant different in the magnesium level were recorded in the modern group between Day 90 and Day 180 (P < 0.05) and in the lifestyle group between Day 90 and Day 180 (P < 0.05) (Table 6 & Fig. 3). Statistically significant group interaction was also recorded between the control and modern groups (P < 0.05) (Table 5). On the other hand, the magnesium levels in the prediabetic control and trial groups did not recorded any significant changes over the different day-wise intervals. Furthermore, the interaction between prediabetic control and trial groups did not resulted in any variations in magnesium levels (Table 6 & Fig. 4). Although no statistical significance was recorded, it is important to note that all groups-maintained magnesium levels within the normal range throughout the study period (Table 5).

##### 3.1.2.6 Copper

The copper levels remained relatively stable in the control and AYUSH groups over the day-wise intervals (Table 6 & Fig. 3). However, statistically significant differences were observed in the modern group between Day 0 and Day 180 (P < 0.05) and between Day 90 and Day 180 (P < 0.05). The lifestyle group also displayed a statistically significant difference between Day 90 and Day 180 (P < 0.05) (Table 6 & Fig. 3). Statistically significant interactions were recorded between the control and modern groups (P < 0.05), control and AYUSH groups (P < 0.05), control and lifestyle groups (P < 0.05), modern and lifestyle groups (P < 0.05) and AYUSH and lifestyle groups (P < 0.05) (Table 5). In the prediabetic trial group, copper level slightly increased from Day 0 (119.5 ± 25.6) to Day 90 (123.9 ± 25.9) (P = 0.4109) and Day 180 (125.5 ± 18) (P = 0.1694) (Table 5, Table 6 & Fig. 4). Whereas the group comparison recorded a statistically significant difference in copper levels in prediabetic control and trial group (P < 0.05) (Table 5).

##### 3.1.2.7 Calcium

The calcium levels continued to be steady across all treatment groups over the different day-wise intervals (Table 6 & Fig. 3). The interactions between the groups, including control vs modern, control vs AYUSH, control vs lifestyle, modern vs AYUSH, modern vs lifestyle, and AYUSH vs lifestyle were also not significantly different (Table 5). Furthermore, there was no significant variation in calcium levels between the prediabetic control and prediabetic trial groups (Table 5, Table 6 & Fig. 4).

#### 3.1.3 Impact of DiabetEaze powder on glycemic outcomes

##### 3.1.3.1 FBS

**Fig: 5:**
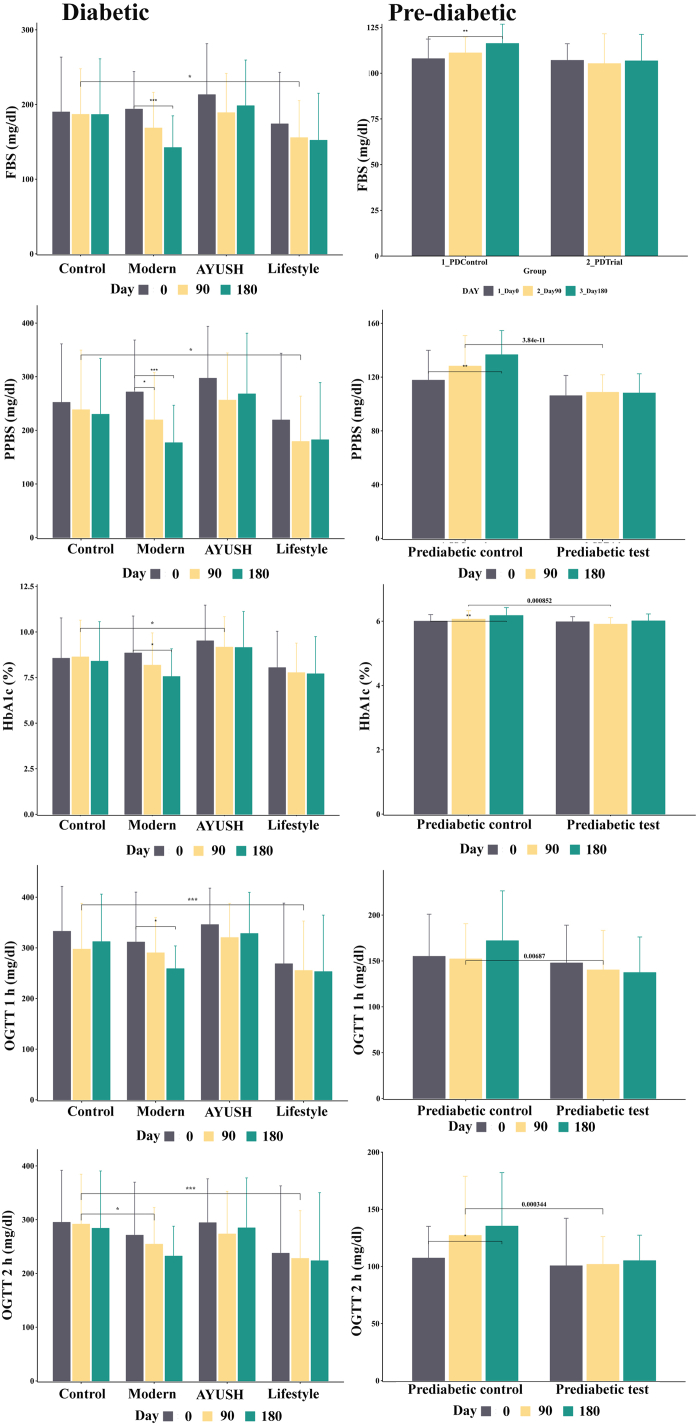
Glycemic response in the treatment groups to DiabetEaze powder. Error bars represent SEM.

The study assessed FBS levels in all treatment groups before and after DiabetEaze powder treatment (Table 7 & Fig. 5). The FBS levels in the control group decreased from Day 0 to Days 90 and 180, though the differences were not statistically significant. The modern group had significantly lower FBS levels at Day 0, 90, and 180 (P < 0.05). Similarly, the AYUSH group also recorded significantly lower FBS levels from Day 0 to Day 90 (P < 0.05). In case of lifestyle group, significantly lower FBS levels was recorded on Day 0 vs. 90 (P < 0.05) (Table 7 & Fig. 5). Statistically significant differences in the FBS level were recorded between the control vs. lifestyle group (P < 0.05), modern vs. AYUSH group (P < 0.05), and AYUSH vs. lifestyle group (P < 0.05) (Table 5). Significant variation in FBS level were recorded between the in the prediabetic control and trial groups (P < 0.05) (Table 5, Table 7 & Fig. 5).

**Table 7:** Day-wise comparative analysis of glycemic index levels across treatment groups following administration of DiabetEaze powder.

Variables	Groups	Day 0 Mean ± SD	Day 180 Mean ± SD	Day 90 Mean ± SD	Significant difference between Days		
					Day 0 Vs 90	Day 0 Vs 180	Day 90 Vs 180
HbA1c (%)	Control	8.6 ± 2.2	8.6 ± 2	8.4 ± 2.2	P = 0.779	P = 0.595	P = 0.276
	Modern	8.8 ± 2	8.2 ± 1.8	7.6 ± 1.5	P ≤ 0.001	P ≤ 0.001	P ≤ 0.001
	AYUSH	9.5 ± 2	9.2 ± 1.7	9.1 ± 2	P = 0.088	P = 0.090	P = 0.896
	Lifestyle	8 ± 2	7.8 ± 1.6	7.7 ± 2	P = 0.148	P = 0.268	P = 0.771
	Prediabetic control	6 ± 0.21	6.18 ± 0.25	6.06 ± 0.26	P = 0.042	P<0.001	P = 0.013
	Prediabetic Trial	5.98 ± 0.16	6.01 ± 0.22	5.91 ± 0.21	P = 0.059	P = 0.373	P = 0.033
OGTT (1 h) (mg/dl)	Control	345.9 ± 72	328.3 ± 81.3	320.3 ± 67.5	P = 0.068	P = 0.224	P = 0.303
	Modern	271 ± 98.7	232.2 ± 55.6	254.3 ± 68.3	P = 0.106	P ≤ 0.001	P ≤ 0.001
	AYUSH	345.9 ± 72	328.3 ± 81.3	320.3 ± 67.5	P = 0.033	P = 0.183	P = 0.412
	Lifestyle	268.6 ± 119.7	253.1 ± 111.5	255.3 ± 97.7	P = 0.436	P = 0.251	P = 0.873
	Prediabetic control	155 ± 46	172 ± 54.4	152.2 ± 38.4	P = 0.675	P = 0.040	P = 0.007
	Prediabetic Trial	147.8 ± 41.2	137.3 ± 38.8	140.2 ± 43.1	P = 0.201	P = 0.051	P = 0.502
OGTT (2 h) (mg/dl)	Control	295 ± 96.8	291 ± 123.8	291.6 ± 92.8	P = 0.807	P = 0.841	P = 0.963
	Modern	271 ± 98.7	232.2 ± 55.6	254.3 ± 68.3	P = 0.208	P = 0.019	P = 0.001
	AYUSH	294.3 ± 81.7	284.7 ± 93	273.3 ± 79.3	P = 0.467	P = 0.068	P = 0.317
	Lifestyle	237.5 ± 125.4	223.5 ± 126.7	227.9 ± 88.8	P = 0.625	P = 0.486	P = 0.809
	Prediabetic control	107.3 ± 27.8	135.3 ± 46.8	127.1 ± 51.8	P = 0.086	P = 0.007	P = 0.357
	Prediabetic Trial	100.6 ± 41.6	105 ± 22.3	101.8 ± 24.3	P = 0.572	P = 0.884	P = 0.388
FBS (mg/dl)	Control	190 ± 73.5	186.6 ± 74.6	186.8 ± 61.1	P = 0.707	P = 0.785	P = 0.985
	Modern	193.8 ± 50.3	168.5 ± 47.8	142.3 ± 42.5	P ≤ 0.001	P ≤ 0.001	P ≤ 0.001
	AYUSH	213.2 ± 68.3	189.1 ± 52.6	198.3 ± 61.2	P = 0.007	P = 0.066	P = 0.131
	Lifestyle	174 ± 69.1	155.6 ± 49.6	152.1 ± 63	P = 0.025	P = 0.059	P = 0.663
	Prediabetic control	107.9 ± 10.7	116.2 ± 10.6	111.1 ± 8.7	P = 0.062	P ≤ 0.001	P = 0.005
	Prediabetic Trial	107 ± 9.1	106.7 ± 14.5	105.3 ± 16.3	P = 0.411	P = 0.878	P = 0.487
PPBS (mg/dl)	Control	252.13 ± 109.13	271.73 ± 97.51	298.7 ± 100.58	P = 0.40	P = 0.744	P = 0.704
	Modern	221.2 ± 129.8	245.03 ± 128.74	176.87 ± 69.87	P ≤ 0.001	P ≤ 0.001	P ≤ 0.001
	AYUSH	267.95 ± 113.2	188.7 ± 130.3	238.37 ± 111.24	P = 0.005	P = 0.040	P = 0.341
	Lifestyle	219.4 ± 88.37	256.27 ± 88.06	179.17 ± 84.59	P = 0.008	P = 0.159	P = 0.525
	Prediabetic control	117.7 ± 22.3	136.6 ± 18	128.2 ± 22.7	P = 0.050	P ≤ 0.001	P = 0.038
	Prediabetic Trial	106.2 ± 15	108.2 ± 14.3	108.7 ± 13	P = 0.359	P = 0.554	P = 0.846

##### 3.1.3.2 HbA1c

One of the major primary efficacy parameters evaluated in this clinical trial was the variation in HbA1c levels from Day 0 to Day 180 across all treatment groups (Table 7 & Fig. 5). At Day 0, HbA1c level of diabetic control group is 8.6 ± 2.2, prediabetic control group is 6 ± 0.2, modern group is 8.8 ± 2, AYUSH group is 9.5 ± 2, lifestyle group is 8 ± 2 and prediabetic trial group is 5.98 ± 0.16. At Day 180, HbA1c levels reduced to 8.4 ± 2.2 in the control group, 7.6 ± 1.5 in the modern group, 9.1 ± 2 in the AYUSH group, 7.7 ± 2 in the lifestyle group, and 5.91 ± 0.21 in the prediabetic trial group. Even though reduction in the HbA1c displayed, statistically analysis revealed no significant reduction in HbA1c levels in the control group from Day 0 to Day 180. In case of modern group, a significant reduction in HbA1c levels from Day 0 (8.8 ± 2) to Day 90 (8.2 ± 1.8) and Day 180 (7.6 ± 1.5) (Table 7 & Fig. 5). The HbA1c levels in the AYUSH and lifestyle groups did not differ significantly over the course of the study, indicating that these groups displayed same efficacy. In group wise analysis, significant differences in the interactions were recorded between the AYUSH vs. lifestyle (P < 0.05), control vs. lifestyle, and modern vs. AYUSH groups (Table 5). Whereas control vs. modern and modern vs. lifestyle groups were not statistically significant. In addition to this, there was no significant variation in HbA1c levels was recorded between the prediabetic control and trial groups (P < 0.05) (Table 5, Table 7 & Fig. 5).

##### 3.1.3.3 Effect on OGTT level

The table recorded the level of OGTT in the treatment groups before and after treatment with DiabetEaze powder (Table 7 & Fig. 5). In the control and lifestyle groups, after 90 and 180 days of intervention, the changes in OGTT in the first hour and second hour were not statistically significant. In the modern group, Day 0 vs. 180 and Day 90 vs. 180 recorded a significant difference; likewise, the AYUSH group also recorded a significant difference between Day 0 vs. 90. Interactions between the control vs. modern, control vs. lifestyle, modern vs. AYUSH, modern vs. lifestyle, AYUSH vs. lifestyle, prediabetic control vs. prediabetic trial (P < 0.05) at hour 1 and 2 were significantly different except control vs. AYUSH (Table 5 & Fig. 5).

##### 3.1.3.4 PPBS

The PPBS levels recorded variation in the tested groups at different day intervals. In the control group, PPBS levels did not change significantly over time (Table 7 & Fig. 5). The modern group showed a significant reduction in PPBS levels from Day 0 to Day 90 (P < 0.05). The AYUSH group also experienced significant reductions in PPBS levels from Day 0 to Day 90 (P < 0.05) and Day 180 (P < 0.05). On Day 90, the lifestyle group had a significant decrease in PPBS levels (P < 0.05), followed by an increase on Day 180 with no significant difference. Significant differences were recorded between the control and lifestyle groups (P < 0.05), modern and AYUSH groups (P < 0.05), and AYUSH and lifestyle groups (P < 0.05) (Table 5). In case of prediabetic control group, PPBS levels increased significantly from Day 0 to Day 90 and Day 180 (P < 0.05) (Table 5, Table 7 & Fig. 5). However, no significant variation in PPBS levels was found in the prediabetic trial group across different day intervals. Whereas the group wise interaction showed that the PPBS levels between the prediabetic control and trial groups recorded significant effect (P < 0.05) (Table 5, Table 7 & Fig. 5).

### 3.2 QOL

The overall mean QOL score increase in the modern, lifestyle, AYUSH and prediabetic groups when related to the respective control groups after the administration of DiabetEaze powder (Table 8). In conclusion, the administration of nutritional formulas tailored to the specific needs of diabetic patients contributed to a significant improvement in QoL. Furthermore, limitation due to physical health, physical endurance, diet satisfaction, general health, etc. enhanced in the treatment groups when compared to the control.

**Table 8:** Day-wise comparative analysis of QoL across treatment groups following administration of DiabetEaze powder.

Variables	Groups	Day 0	Day 90	Day 180	Significant difference between		
		Mean ± SD	Mean ± SD	Mean ± SD	Day 0 Vs 90	Day 0 Vs 180	Day 90 Vs 180
Role Limitation Due to Physical Health	Control	24.34 ± 1.737	24.2 ± 1.780	23.3 ± 1.674	P = 1	P ≤ 0.001	P = 0.012
	Modern	24.7 ± 1.553	26.6 ± 1.520	28.48 ± 1.5889	P ≤ 0.001	P ≤ 0.001	P ≤ 0.001
	AYUSH	25.57 ± 1.937	26.7 ± 1.577	28.45 ± 1.655	P ≤ 0.001	P ≤ 0.001	P ≤ 0.001
	Lifestyle	25.3 ± 1.582	26.0 ± 1.494	28.4 ± 1.088	P ≤ 0.001	P ≤ 0.001	P ≤ 0.001
	Prediabetic control	25.4 ± 1.832	25.2 ± 1.769	25.8 ± 1.510	P = 0.465	P = 1	P = 1
	Prediabetic Trial	26 ± 1.244	27 ± 1.455	28 ± 1.489	P ≤ 0.001	P ≤ 0.001	P = 0.003
Physical Endurance	Control	24.6 ± 1.785	24.4 ± 1.824	24.2 ± 2.411	P = 1	P = 1	P = 1
	Modern	25 ± 2.456	26 ± 2.176	27.1 ± 1.893	P = 0.007	P ≤ 0.001	P = 0.001
	AYUSH	25.1 ± 3.014	26.2 ± 2.678	27.0 ± 2.710	P<0.001	P ≤ 0.001	P = 0.127
	Lifestyle	25.5 ± 1.877	25.9 ± 1.628	26.9 ± 1.436	P = 1	P ≤ 0.001	P = 0.004
	Prediabetic control	25.5 ± 1.814	25.5 ± 1.776	25.7 ± 1.749	P = 1	P = 1	P = 1
	Prediabetic Trial	25.6 ± 2.089	26.3 ± 1.949	27.3 ± 1.922	P = 0.587	P ≤ 0.001	P = 0.003
General Health	Control	12.3 ± 0.930	12.3 ± 0.922	12.1 ± 1.187	P = 1	P = 1	P = 1
	Modern	12.5 ± 1.179	13.1 ± 1.391	13.5 ± 0.995	P = 0.106	P ≤ 0.001	P = 0.006
	AYUSH	12.6 ± 1.458	13.1 ± 1.391	13.5 ± 1.371	P = 0.027	P ≤ 0.001	P ≤ 0.001
	Lifestyle	12.7 ± 0.902	12.9 ± 0.870	13.4 ± 0.807	P = 1	P ≤ 0.001	P = 0.017
	Prediabetic control	12.6 ± 0.928	12.8 ± 0.817	12.9 ± 1.819	P = 1	P = 1	P = 1
	Prediabetic Trial	12.7 ± 0.987	13.0 ± 0.954	13.6 ± 0.973	P = 1	P ≤ 0.001	P ≤ 0.001
Treatment Satisfaction	Control	16.6 ± 1.402	16.4 ± 1.323	16.3 ± 1.583	P = 1	P = 1	P = 1
	Modern	16.8 ± 1.746	17.3 ± 1.469	18.1 ± 1.284	P = 0.347	P ≤ 0.001	P = 0.001
	AYUSH	16.7 ± 2.066	17.5 ± 1.906	18.2 ± 1.867	P = 0.002	P ≤ 0.001	P = 0.005
	Lifestyle	17.0 ± 1.354	17.4 ± 1.230	17.9 ± 1.012	P = 1	P ≤ 0.001	P = 0.618
	Prediabetic control	17 ± 1.217	16.9 ± 1.258	171 ± 1.242	P = 1	P = 1	P = 1
	Prediabetic Trial	17.1 ± 1.430	17.5 ± 1.314	18.3 ± 1.400	P = 1	P ≤ 0.001	P = 0.004
Treatment Satisfaction	Control	12.3 ± 0.930	12.3 ± 0.922	12.1 ± 1.187	P = 1	P = 1	P = 1
	Modern	12.5 ± 1.179	13.0 ± 1.033	13.5 ± 0.995	P = 0.106	P ≤ 0.001	P = 0.006
	AYUSH	12.6 ± 1.458	13.1 ± 1.391	13.5 ± 1.371	P = 0.027	P ≤ 0.001	P = 0.027
	Lifestyle	12.6 ± 1.458	12.9 ± 0.870	13.4 ± 0.807	P = 1	P ≤ 0.001	P = 0.017
	Prediabetic control	12.6 ± 0.928	12.8 ± 0.817	12.9 ± 0.819	P = 1	P = 1	P = 1
	Prediabetic Trial	12.7 ± 0.987	13 ± 0.954	13.6 ± 0.973	P = 1	P ≤ 0.001	P ≤ 0.001
Financial Worries	Control	15.8 ± 1.215	15.7 ± 1.137	16.5 ± 1.526	P = 1	P = 1	P = 1
	Modern	16.0 ± 1.581	16.5 ± 1.362	17.3 ± 1.210	P = 0.084	P ≤ 0.001	P = 0.004
	AYUSH	15.8 ± 2.027	16.6 ± 1.692	17.3 ± 1.744	P ≤ 0.001	P ≤ 0.001	P = 0.003
	Lifestyle	16.1 ± 1.231	16.5 ± 1.122	17.0 ± 1.016	P = 1	P ≤ 0.001	P = 0.167
	Prediabetic control	16.1 ± 1.125	16.2 ± 1.095	16.3 ± 1.055	P = 1	P = 1	P = 1
	Prediabetic Trial	16.3 ± 1.336	16.6 ± 1.193	17.4 ± 1.262	P = 1	P ≤ 0.001	P ≤ 0.001
Emotional/Mental Health	Control	20.5 ± 1.526	20.4 ± 1.476	20.2 ± 1.971	P = 1	P = 1	P = 1
	Modern	20.9 ± 2.071	21.7 ± 1.770	22.6 ± 1.585	P = 0.013	P ≤ 0.001	P = 0.007
	AYUSH	20.8 ± 2.551	21.8 ± 2.194	22.6 ± 2.289	P = 0.002	P ≤ 0.001	P = 0.003
	Lifestyle	21.2 ± 1.499	21.6 ± 1.409	22.4 ± 1.283	P = 1	P ≤ 0.001	P = 0.024
	Prediabetic control	21.2 ± 1.341	21.2 ± 1.400	21.4 ± 1.377	P = 1	P = 1	P = 1
	Prediabetic Trial	21.3 ± 1.659	21.9 ± 1.711	22.7 ± 1.651	P = 0.656	P ≤ 0.001	P = 0.003
Diet Satisfaction	Control	11.7 ± 1.023	12.0 ± 0.997	12.2 ± 0.901	P = 1	P = 1	P = 1
	Modern	11.87 ± 2.320	13.12 ± 1.0763	14.2 ± 0.773	P ≤ 0.001	P = 1	P ≤ 0.001
	AYUSH	12.58 ± 1.359	13.57 ± 1.00	13.9 ± 1.079	P ≤ 0.001	P ≤ 0.001	P = 1
	Lifestyle	12.6 ± 0.877	13.3 ± 0.908	14.5 ± 0.567	P = 0.154	P ≤ 0.001	P ≤ 0.001
	Prediabetic control	11.9.6 ± 0.739	12.3 ± 0.749	12.3 ± 0.758	P = 1	P = 1	P = 1
	Prediabetic Trial	13 ± 0.936	13.0 ± 0.780	15 ± 0.466	P = 0.036	P ≤ 0.001	P ≤ 0.001

### 3.3 Safety profile

#### 3.3.1 Hematology, lipid & electrolyte profile

Hematology, serum lipid and electrolyte levels were measured in both groups before and after the study. Table 9 shows no significant differences in hematology, serum lipid and electrolyte levels between treatment groups and the control group on the first and last day.

**Table 9:**
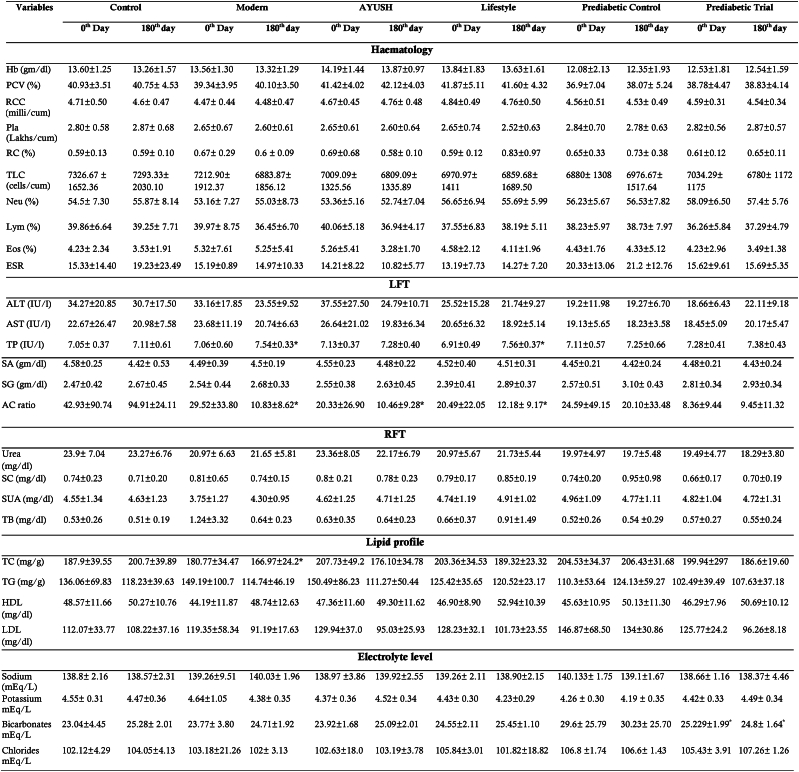
Hematology, LFT, RFT, lipid profile and electrolyte level in the subjects before and after the administration of DiabetEaze powder. ∗p < 0.05

HB-Haemoglobin, PCV-Packed Cell Volume, RCC-Red Cell Count, RC- Reticulocyte Count, TLC-total leucocyte count, ALT- Alanine aminotransferase, AST-aspartate aminotransferase, ALP - alkaline phosphatase, TP- total protein, SA - serum albumin, SG-serum globulin, AC Ratio - albumin creatinine ratio, SC- serum creatinine, SUA-serum uric acid, TB- total bilirubin, TC- total cholesterol, TG-triglyceride, HDL-high-density lipoprotein, LDL-low-density lipoprotein cholesterol, Pla-platelet, Neu-neutrophils, Lym-lymphocytes, Eos = eosinophils.

#### 3.3.2 LFT/RFT

The outcome from the study clearly showed that there was no significant difference in the mean LFT and RFT values of all variables between the initial and final time points of intervention. Furthermore, the LFT and RFT values in all treatment groups were clinically within the normal range for a healthy person, indicating that the trial subject's liver and kidney functions were not adversely affected due to the administration of DiabetEaze powder (Table 9). Thus, it was determined that DiabetEaze powder is not hepatic/renal toxic and is safe to use at the recommended dosage.

### 3.4 Safety and adverse effect

Subjects did not report any significant side effects. However, the treatment group reported two cases of cramps during the first few days of the study. DiabetEaze powder was found to be both safe and well tolerated in this study. DiabetEaze powder was also well tolerated, with no participant reporting any side effects other than a few minor gastrointestinal issues.

## 4 Discussion

DM is a chronic metabolic disorder that has attained epidemic status around the globe [56]. According to the IDF reports, in 2021, more than 530 million adults worldwide were living with diabetes. In 2023, Anjana [5] and her co-worker published their findings in a Lancet article stating that India had around 77 million adults living with diabetic and nearly 25 million prediabetic conditions [5]. The prevalence rate of DM in India is around 8.8 %, with a substantial portion being undiagnosed [57]. Nowadays, diabetic people are extremely susceptible to various nutrient deficiencies, predominantly minerals and vitamins [12]. As the diabetes epidemic continues, confirming proper nutrient intake by individuals remains an important challenge [58]. A variety of interconnected factors also contribute to nutrient deficiencies in diabetic people. First, high blood sugar levels may cause an increase in the urinary excretion of essential nutrients such as magnesium, zinc, and chromium. Second, diabetic neuropathy and gastroparesis can interfere with nutrient digestion and absorption, aggravating deficiencies of various nutrients [12]. Furthermore, dietary restrictions and food choices intended to regulate blood sugar can occasionally result in an inadequate supply of essential nutrients for the body. Finally, some diabetes drugs, for example, metformin, can restrict nutrient absorption or cause nutrient loss. In order to overcome this issue, diabetic people, particularly the elderly, must consume a balanced and nutrient-rich diet [59]. As a result, regular nutrient level monitoring and a well-balanced diet or nutrient supplements are critical for managing diabetes and avoiding related nutrient deficiencies [27]. Additionally, nutrient supplements recommended for diabetics, particularly the elderly, may be required to maintain adequate nutrient levels to overcome the above-mentioned issues. Multiple small-scale studies reported that more than half of elderly diabetic patients may be affected by malnutrition [60]. Also, the rate of undernutrition, as Tamura et al., 2020 [60] assessed by the Mini Nutritional Assessment (MNA), appears to be higher in older adults with diabetes compared to those without diabetes. A detailed survey conducted in Spain involving 1,090 hospitalized older patients with diabetes revealed that 39.1 % were at risk of malnutrition, while 21.2 % had confirmed malnutrition [60]. These results highlight the importance of screening and properly addressing malnutrition in elderly adults with diabetes to optimize their health outcomes and QoF. In the present study, a six-arm randomized, open-label, comparative, multi-center, investigator-initiated clinical trial was conducted to investigate the effects of DiabetEaze Power, an polyherbal nutritional formulation, on nutritional and glycemic management in T2D and prediabetic patients.

Poorly controlled DM patients were highly prone to micronutrient deficiencies; hence, it is vital to fulfil vitamins and minerals through food according to their needs [12]. Besides, most diabetic people, especially elderly people, will not get sufficient nutrients from the diet alone. Deficiencies in micronutrients in diabetic people lead to various metabolic syndromes [60]. Micro-minerals such as phosphorus, potassium, and magnesium are complicated in diverse components of the metabolic syndrome, especially in the secretion of insulin [61]. Additionally, low magnesium levels could result in impaired insulin action as well as altered cellular glucose transport [62]. Additionally, micronutrient deficiencies such as zinc, chromium, magnesium, copper, and manganese are associated with glucose intolerance [63,64]. Many studies have confirmed the benefits of micronutrients for diabetics. Likewise, the metabolic abnormalities associated with diabetes can increase the body's demand for certain nutrients. For instance, insulin resistance and hyperglycemia may elevate the requirement for microminerals like magnesium, zinc, chromium and antioxidants like vitamins C and E [12]. In our study, patients diagnosed with diabetes and prediabetes were administered with DiabetEaze powder for nutritional management and diabetic control. The study revealed that DiabetEaze powder recorded varied patterns of micromineral levels across different intervention groups. Besides, the administration of DiabetEaze powder in diabetic and prediabetic patients's recorded significant increases in the level of certain microminerals. In addition, the enhancement in the micromineral varies depending up on the treatment groups.

Uncontrolled hyperglycaemia, especially in those on chronic diuretic therapy, is prone to developing deficiencies in some minerals, notably potassium, magnesium, and zinc [12]. Deficiencies of certain minerals, such as potassium, magnesium, zinc and chromium may lead to carbohydrate intolerance. These micro- and macro-nutrients play precise roles in the pathogenesis and progression of T2D and the mechanisms of action of some of the other major macro- and micro-elements are altered in T2D [65]. Patients with uncontrolled diabetes have increased zinc losses through urine. Generally, these losses were balanced by enhanced zinc absorption via the gut [66,67] It is also essential for the synthesis, storage, and secretion of insulin and acts as a structural component of hormones that ensures proper functioning [42]. Zinc also helps in the production and release of insulin from the pancreas and enhances insulin sensitivity and glucose uptake by cells, particularly those in muscle and adipose tissues. It was earlier reported that zinc insufficiency has been linked in certain studies to decreased insulin production and increased tissue resistance to the effects of insulin [68]. In the present study, stable zinc levels were recorded in all the intervention groups. Further, the intervention groups recorded improvement in their glycemic parameters, and this may be due to the enhanced insulin production and glucose uptake by the cells due to the stable level of serum zinc.

Magnesium also plays a vital role in maintaining blood glucose levels, contributing to overall metabolic health. Magnesium is a silent hero in the realm of metabolic health, employing a positive effect on blood glucose levels at cellular levels [69]. As a cofactor for enzymes involved in the insulin signaling pathway, it enhances the sensitivity of cells to insulin [70]. This enhanced sensitivity enables effective glucose uptake and thus reduces the risk of insulin resistance. In our study, significant differences in manganese levels were recorded in the modern, AYUSH, and lifestyle groups. Additionally, these groups showed noteworthy improvement in the glycemic parameters, which may be due to the positive influence of magnesium on maintaining blood glucose levels.

Selenium, another essential micronutrient, plays structural and enzymatic roles in antioxidant defense systems [70]. In diabetic conditions, oxidative stress increases and causes cellular damage. Selenium helps in the formation and activation of antioxidant enzymes, mainly glutathione peroxidase, which neutralizes ROS and free radicals at the cellular level, as well as other harmful free radicals, thus protecting cells from oxidative stress and averting further damage [71]. In our study, selenium levels increased in all treatment groups except prediabetics. The enhanced level of selenium in the serum may be efficiently used at tissue levels to overcome oxidative stress. The selenium present in the DiabetEaze powder may effectively neutralize free radicals, improve insulin sensitivity, and protect mitochondria and pancreatic β-cells. Overall, these functions improve glucose metabolism and help to maintain blood sugar levels within a normal range. The selenium in the formulation appears to be effectively used by the body to lower blood glucose levels and combat stress. Unlike the control group, the intervention groups that received DiabetEaze powder experienced a significant decrease in blood glucose levels, demonstrating the formulation's efficacy. This demonstrates the complex relationship between formulation design and molecular interactions in the body. DiabetEaze powder has the potential to be an effective tool for managing blood glucose levels by increasing selenium bioavailability and exploiting its critical role in glucose metabolism.

The rising prevalence of DM has been linked not only to deficiencies in macro- and microminerals but also to a deficiency in key vitamins. Studies suggest that vitamins A, C, D, E, and B-vitamins play a crucial role in the pathogenesis of diabetes [72,73]. Furthermore, metformin, a widely used antidiabetic drug, has been associated with reduced absorption of vitamin B12, leading to vitamin B12 deficiency over time. This deficiency can further complicate the management of diabetes. The present study was a randomized, six-arm, open-labelled clinical trial involving 211 T2D and prediabetic patients. These patients were divided into six groups—modern, AYUSH, lifestyle, and prediabetic—with 30 patients in each group. Over six months, our study found that vitamin B12 levels remained stable across day-wise intervals in all groups. This is consistent with earlier research by Satapathy et al. [74], who conducted a randomized, multi-arm, open-labelled clinical trial involving 80 T2D patients. In that study, patients were divided into four groups of 20 each and observed over 8 weeks. Their results confirmed that supplementation with vitamin B12 improved both glycaemic control and insulin resistance in T2D patients. Furthermore Elbarbary et al. [75] reported that a 12-week supplementation with vitamin B complex in the intervention group significantly reduced fasting blood glucose, and HbA1c. In our study, interactions between the groups showed significant differences in vitamin B12 levels, suggesting that vitamin B12 supplementation through the DiabetEaze powder effectively improved glycaemic parameters. This aligns with the findings of previous research, which showed that B-vitamin supplementation can alleviate neuropathic symptoms and improve glucose metabolism in diabetic patients [76,77]. Components of DiabetEaze powder, such as *Phyllanthus emblica*, *Moringa oleifera*, and *Artocarpus heterophyllus*, have been shown to enhance B-vitamin content and contribute to improved glucose regulation [41]. Our findings support these studies, suggesting that DiabetEaze powder may help address B-vitamin deficiencies and improve neuropathic symptoms in T2D patients. The positive impact of DiabetEaze powder on B-vitamin levels in our study is consistent with the work of Sapkota et al. [35], who observed improvements in vitamin B12 and folate levels following the use of herbal formulations in diabetic patients. These improvements were clearly reflected in the glycaemic parameters of the various intervention groups in our study. Overall, the results of our study provide further evidence that DiabetEaze powder can be beneficial in improving both B-vitamin status and glycaemic control in T2D patients.

Diabetic conditions are often characterized by chronic low-grade inflammation, which can increase the body's demand for antioxidant vitamins such as Vitamin C and E, potentially leading to reduced levels of these essential micronutrients. In addition, diabetes-related complications, including nephropathy, retinopathy, and neuropathy, can further influence nutrient status. For instance, kidney dysfunction in diabetic nephropathy impairs the body's ability to regulate electrolyte balance and excrete waste products, leading to altered vitamin D metabolism and other mineral imbalances. Scientific research has highlighted the crucial roles of calcium and vitamin D, not only in maintaining skeletal health but also in immune modulation and regulating insulin secretion by the pancreas [78,79]. Studies have shown that vitamin D supplementation in prediabetic patients can reduce the incidence of T2D and accelerate the transition from prediabetes to normoglycemia [80]. Previous research by Li et al. [81] demonstrated that vitamin D supplementation reduces insulin resistance in patients with T2D, a critical factor in glycemic management. Cojic et al. [82] further reported that daily oral administration of vitamin D significantly reduced HbA1c levels within a treatment period of three to six months. Similarly, Aljabri et al. [83] observed improved glycemic control in individuals with type 1 diabetes mellitus who were vitamin D deficient following supplementation. Supporting these findings, Chen et al. [84] highlighted that vitamin D supplementation significantly decreased fasting blood glucose (FBG), HbA1c, homeostatic model assessment for insulin resistance (HOMA-IR), and fasting insulin levels in T2D patients. A randomized controlled trial by Nikooyeh et al. [85] also demonstrated a substantial reduction in HbA1c levels following vitamin D administration compared to a placebo. More recently, Thenmozhi et al. [86] demonstrated that vitamin D supplementation effectively brought FBG and HbA1c levels within the normal clinical range, thereby potentially mitigating complications associated with DM. These findings collectively underscore the potential of vitamin D as an adjunctive therapeutic agent for improving glycemic control and reducing diabetes-associated risks. In line with these findings, the present study demonstrated that six months of continuous administration of DiabetEaze powder significantly enhanced vitamin D levels across all treatment groups. Furthermore, the treatment groups exhibited lower HbA1c levels, which may be attributed to the vitamin D provided by the DiabetEaze powder. These results suggest that supplementation with DiabetEaze powder may have a beneficial role in enhancing vitamin D status and improving glycemic control in patients with T2D and prediabetes. The findings highlight the potential of the herbal components of DiabetEaze powder in addressing two critical aspects of diabetes management: correcting vitamin D deficiency and mitigating insulin resistance, a key pathogenic factor in T2D and prediabetic states. This dual-action mechanism positions DiabetEaze powder as a promising adjunctive therapeutic option for improving metabolic outcomes in these populations.

The nutritional profile of Pankajakasthuri DiabetEaze powder, as evidenced in this study, highlights its potential in addressing malnutrition-associated challenges in T2D and prediabetic patients. DiabetEaze powder provides a balanced macronutrient profile with moderate protein content (10.70 g/100 g), low-fat levels (6.72 g/100 g), and high fiber (12.95 g/100 g). Dietary fiber plays a critical role in regulating postprandial glycemic excursions by slowing carbohydrate absorption and enhancing insulin sensitivity. Furthermore, the absence of sucrose aligns with the dietary recommendations for diabetics, mitigating the risk of hyperglycemia while promoting steady energy release, as reflected in its energy value of 389.36 kcal/100 g. Micronutrient deficiencies, particularly in vitamins and minerals, are often overlooked in diabetic management but have substantial implications for glucose metabolism. DiabetEaze powder's enrichment with essential vitamins, including B-complex vitamins (B1, B2, B6, B12, and B9), vitamin D, and vitamin C, addresses critical deficits that exacerbate diabetic complications. Minerals such as zinc, magnesium, manganese, and chromium further complement its efficacy. Zinc and chromium are critical for insulin synthesis and action, while magnesium modulates glucose uptake and insulin signaling. The observed stability or enhancement of these micronutrient levels across treatment groups underscores the formulation's capability to rectify subtle nutritional deficiencies that are often prevalent in diabetic populations due to dietary restrictions or metabolic derangements. This balanced nutritional profile of DiabetEaze powder not only optimizes metabolic pathways but also contributes to improved glycemic indices observed in the trial. Notably, elevated fiber, coupled with critical micronutrient support, is particularly beneficial for prediabetic patients, as seen in significant group-wise improvements in biochemical parameters. The inclusion of DiabetEaze powder as part of the dietary regimen for T2D and prediabetic patients provides a dual advantage: addressing the nutritional deficiencies that impair glucose homeostasis and enhancing compliance through its palatable formulation. These findings align with global efforts to integrate nutritional therapy into diabetes management, reducing reliance on pharmacological interventions and improving patient quality of life.

The current study also investigated at the effect of DiabetEaze powder on glycemic parameters in diabetics and prediabetics. Significant reductions in fasting blood sugar levels were seen in the intervention groups, particularly in AYUSH and lifestyle. These groups also had significantly lower postprandial blood sugar levels on the 90th and 180th days, whereas the modern trial group only showed reductions on the 180th day. The OGTT results showed a significant drop on the 180th day for the lifestyle group and on the 90th and 180th days for the AYUSH group. Furthermore, HbA1C levels decreased significantly on the 180th day across all trial groups. The key ingredient of DiabetEaze powder, *C. longa*, rich in curcumin and related compounds, has been widely investigated for its anti-diabetic effects [87]. Its ability to reduce FBG and HbA1C levels is well documented. Additionally, the formulation's other components, including *E. officinalis, M. pudica, S. potatorum*, and others, contribute to its antidiabetic potential through various mechanisms, such as regulating digestive enzymes, enhancing insulin sensitivity, and reducing oxidative stress [[31], [32], [33], [34], [35], [36], [37]]. *E. officinalis* contains ellagic acid and ascorbic acid, which reduce blood glucose levels by inhibiting digestive enzymes like amylase and glucosidase. Additionally, its flavonoids may reduce the risk of diabetic neuropathy [88]. *M. pudica* is reported for its antidiabetic and antihyperlipidemic properties, likely due to its alkaloid or flavonoid content. These compounds can regenerate damaged pancreatic β-cells and inhibit the α-glucosidase enzyme [89]. Various other plants like *C. carvi, M. oleifera, A. paniculate, E. coracana, H. vulgare*, and *A. occidentale* are also recognized for their antidiabetic potential through mechanisms such as α-glucosidase inhibition. The improved glycemic parameter in the treatment groups may be due to the impact of these herbals in the DiabetEaze powder formulation. The use of DiabetEaze powder effectively reduces sugar spikes by regulating PPBS and HbA1c levels, which is a critical aspect of its role in diabetes management. Furthermore, the absence of antinutritional factors in the formulation improves its bioavailability and absorption profile, allowing for better therapeutic outcomes.

Malnutrition has been shown to have a negative impact on the elderly's QoL [90]. Insulin resistance is common in older people with DM, where reduced insulin signalling pathways cause reduced protein synthesis and increased protein degradation, contributing to muscle mass loss. T2D is a significant risk factor for functional impairment and mobility restrictions, which coincide with the onset of sarcopenia and frailty [91]. Our findings showed that DiabetEaze powder has the potential to help patients manage their diabetes and improve their overall health. The outcome of our study agrees with the recent investigations, which reported that the oral administration of dapagliflozin among elderly patients with T2D elicited enhancements in HbA1c levels, nutritional status, BMI and QoL [92]. Furthermore, Khattak et al. [93] evaluated the antidiabetic activity of a polyherbal formulation in patients with T2D, demonstrating a significant reduction in HbA1c concentration after 60 days of administration. The majority of the ingredients in the DiabetEaze formulation have already been reported to improve glucose metabolism, stabilize blood sugar levels, and control hunger cues [[94], [95], [96]]. These parameters can help manage appetites and cravings and further resulted in substantial improvements in QOLID scores following DiabetEaze powder supplementation. Over all outcome of the study suggests that patients who manage their diabetes using modern medicines, AYUSH medicines, and lifestyle changes are more satisfied with their treatment, have fewer negative effects on daily activities, and are less concerned about future complications. Additionally, the supplement appears to provide a sense of comfort, peace of mind, and enhanced emotional well-being, allowing patients to be more resilient in dealing with diabetes-related challenges. The detected reduction in thirst scale scores establishes DiabetEaze powder's potential for managing symptoms such as excessive thirst, which is commonly found diabetic patients. Effective blood glucose management and potential improvement in hydration status highlight DiabetEaze powder's all-around benefits in managing diabetes associated complications. To summarize, DiabetEaze powder provides a multifaceted approach to diabetes management by addressing both physiological and psychological aspects, resulting in improved QoF and overall well-being in diabetes patients.

The therapeutic potential of Pankajakasthuri DiabetEaze powder in glycemic control may be attributed to the synergistic effects of its polyherbal ingredients, each contributing through various mechanisms to regulate blood sugar levels and improve metabolic health. *C. longa* (turmeric), with its active compound curcumin, acts through pathways such as inhibition of hepatic glucose production, upregulation of glucose transporters (GLUT2, GLUT3, and GLUT4), and activation of AMP kinase, enhancing insulin secretion and sensitivity [[97], [98], [99]]. *E. officinalis* offers antioxidant and free radical scavenging properties, which may reduce oxidative stress-induced hyperglycemia [100]. *S. potatorum* enhances pancreatic insulin secretion and potentiates its effects, while Salacia reticulata downregulates enzymes involved in gluconeogenesis, effectively lowering fasting glucose levels [34,101]. Carum carvi (caraway) influences glucose utilization in peripheral tissues, inhibits renal glucose reabsorption, and suppresses hepatic glucose production, contributing to long-term glycemic control [102,103]. Mimosa pudica boosts insulin production and regulates glycolytic and gluconeogenic enzyme activity, maintaining glucose homeostasis [104]. Moringa oleifera stimulates GLUT4 expression, inhibits carbohydrate-digesting enzymes, and supports glycolysis, further assisting in glycemic control [105]. Tinospora cordifolia enhances pancreatic β-cell function and endogenous insulin secretion while reducing insulin resistance [106]. Similarly, Andrographis paniculata improves GLUT4 translocation and reduces hepatic gluconeogenesis, thereby supporting efficient glucose uptake [107,108]. Artocarpus heterophyllus (jackfruit) seeds decrease blood glucose by stimulating insulin secretion and reducing intestinal glucose absorption [109]. Eleusine coracana (finger millet) inhibits key enzymes like α-amylase and α-glucosidase, mitigates postprandial hyperglycemia, and supports pancreatic β-cell regeneration [110]. Hordeum vulgare (barley) combats oxidative stress and inhibits carbohydrate-digesting enzymes, further aiding glucose regulation [111] Anacardium occidentale enhances insulin secretion and mitigates oxidative stress-induced complications [112] while Theobroma cacao (cocoa) promotes GLUT4 translocation and activates AMPK pathways, improving glucose uptake and lipid metabolism [113]. These combined actions address multiple aspects of glucose metabolism, including insulin secretion, glucose absorption, hepatic glucose production, and oxidative stress, highlighting the polyherbal formulation's multifaceted approach in managing T2D and prediabetes effectively. Such integrative mechanisms underline the potential of DiabetEaze powder as a comprehensive nutritional intervention for glycemic control.

Finally, no clinically relevant changes were recorded in the hematology, lipid profile, hepatic function, renal function, and electrolytes at the baseline and end of the study in all treatment groups. These results showed that the six-month intervention did not affect anthropometric components such as weight, BMI, and biochemical markers related to kidney and liver function. Hence, it can be concluded that DiabetEaze powder had no adverse effects on kidney and liver function. Liver function tests, such as AST, ALT, and bilirubin levels, were used to evaluate hepatic health and potential hepatotoxicity from the intervention. Throughout the trial, DiabetEaze powder maintained normal LFT parameters in both groups, indicating that it does not cause liver damage or impair liver functioning. This is especially important for diabetics, who are more likely to develop non-alcoholic fatty liver disease (NAFLD). Renal function tests, including serum creatinine and urea levels, were used to assess kidney function and the risk of diabetic nephropathy. The stable renal function parameters in both groups indicate that DiabetEaze powder does not cause nephrotoxicity or worsen renal function in diabetic patients. This finding is significant given the prevalence of diabetic kidney disease (DKD) and the importance of kidney health in diabetes management. Given the increased vulnerability of diabetics to liver and kidney complications, these findings highlight the safety and potential benefits of DiabetEaze powder as a complementary intervention in diabetes management that does not jeopardise hepatic or renal health.

## 5 Conclusions and recommendations

In conclusion, DiabetEaze powder, a novel polyherbal formulation, demonstrates significant potential for managing nutritional status and glycemic control in T2D and prediabetic patients. This formulation effectively maintains micronutrient levels, reduces HbA1c, improves lipid profiles, and enhances quality of life. The strength of this study lies in its comprehensive evaluation of DiabetEaze powder's effects across various therapeutic approaches, including modern medicines, AYUSH therapies, and lifestyle modifications. To our knowledge, this is the first investigation to assess the role of an herbal nutritional formulation in both nutritional and glycemic management of diabetes using diverse treatment modalities, providing valuable insights into its real-world efficacy. The findings suggest that DiabetEaze powder could be integrated into current clinical practice as a safe and effective adjunct to conventional diabetes management strategies. As part of a holistic care approach, it may complement standard pharmacological treatments such as metformin or insulin, as well as lifestyle interventions, to improve glycemic control and address common vitamin and mineral deficiencies observed in diabetic patients. The formulation's ability to regulate key nutritional factors and improve glycemic and lipid parameters makes it a promising addition to dietary recommendations for T2D and prediabetic patients, especially those seeking a more integrative approach to treatment. Given its positive impact across various treatment modalities, DiabetEaze powder may serve as a safe and effective nutritional supplement to enhance overall patient care. Clinicians could consider incorporating it into personalized care plans to support glycemic regulation and optimize metabolic health in patients with T2D or prediabetes.

## 6 Limitations of the current study

The sample size, although statistically sufficient, may not fully capture the diverse demographic and genetic factors affecting diabetes management, limiting the generalizability of the findings to broader populations. The study's follow-up period, capped at 180 days, provided intermediate-term data but was insufficient to evaluate the long-term sustainability of glycemic and nutritional improvements or the prevention of diabetes-related complications. Dietary and lifestyle habits, while monitored through self-reported logs, may have introduced variability due to individual differences in adherence, highlighting the challenge of ensuring uniform compliance across participants. The reliance on self-reported data further accentuates the risk of bias.

Moreover, the trial excluded specific subpopulations, such as individuals with advanced diabetes-related complications, gestational diabetes, or multiple comorbidities, limiting the applicability of results to these groups. The cultural and regional context of the trial may also affect the extrapolation of findings to populations with different dietary habits, healthcare systems, or cultural influences. Lastly, while the preparation of Pankajakasthuri DiabetEaze powder followed stringent Ayurvedic and phytopharmaceutical standards, potential batch-to-batch variability in herbal formulations pose s a challenge for standardization in larger-scale applications. These limitations emphasize the need for future research to address these gaps, including the design of multi-center, double-blinded studies with longer follow-up durations and more diverse populations to validate and extend the findings of this trial.

Future research should focus on exploring the long-term effects of DiabetEaze powder on glycemic and lipid parameters, as well as its potential to prevent diabetes-related complications. Studies with larger and more diverse populations, as well as extended follow-up periods, are needed to confirm these findings and further evaluate its safety and efficacy in broader clinical settings. Additionally, research should aim to assess the formulation's effects in subpopulations with advanced diabetes-related complications, comorbidities, and other specific conditions, which were excluded in the current trial.

## CRediT authorship contribution statement

**Shan Sasidharan:** Project administration, Conceptualization. **Kasthuri Nair A:** Project administration, Methodology, Investigation. **Lekshmi R:** Methodology, Formal analysis. **Arun Visakh Nair:** Project administration, Funding acquisition, Conceptualization. **Sajna SA:** Investigation. **Sandhu G. Joseph:** Methodology, Investigation. **Arjun Chand CP:** Methodology, Investigation. **Sreejith Satheesan:** Methodology, Investigation. **Arun Pratap:** Project administration. **Nishanth Kumar S:** Writing – original draft. **Jerin Paul:** Software, Formal analysis. **Vipin Nair V:** Investigation. **Vijaya R:** Project administration. **Hareendran Nair J:** Funding acquisition, Conceptualization.

## Ethics approval and written informed consent

This study was conducted following the approval of the Independent Ethics Committee (IEC) of Pankajakasthuri Herbal Research Foundation, under the approval numbers IEC/PKHRF/001/04-21 and IEC/PKHRF/002/04-21 (approved on April 8, 2021). Additionally, it was registered in the Clinical Trials Registry of India (CTRI) with the identifier CTRI/2021/04/032956 on April 20, 2021, ensuring transparency and adherence to regulatory standards. Prior to participation, all subjects provided written informed consent, which affirmed their understanding of the study's nature, objectives, procedures, potential risks, and benefits, and their voluntary agreement to partake and allow the publication of the clinical trial data. The study protocols were carefully crafted to protect participants from any harm or infringement of rights throughout the research process, demonstrating a robust commitment to ethical standards and participant safety.

## Data availability statement

The raw data generated and analyzed during this study are not publicly available in any database. However, de-identified raw data can be made available upon reasonable request to the corresponding author, Dr. Shan Sasidharan, for academic and research purposes. All analyzed data supporting the findings of this study are included within the manuscript.

## Consent for publication

Not applicable.

## Declaration of competing interest

The authors declare the following financial interests/personal relationships which may be considered as potential competing interests:Dr. Shan Sasidharan reports financial support was provided by Department of Pharmacology and Pharmacotherapy, Semmelweis University & Pankajakasthuri Ayurveda Medical College & PG Centre, Killy, Kattakada, Thiruvananthapuram, Kerala, India. Dr. Shan Sasidharan reports a relationship with Department of Pharmacology and Pharmacotherapy, Semmelweis University & Pankajakasthuri Ayurveda Medical College & PG Centre, Killy, Kattakada, Thiruvananthapuram, Kerala, India that includes: employment. Dr. Shan Sasidharan has patent nil pending to nil. nil If there are other authors, they declare that they have no known competing financial interests or personal relationships that could have appeared to influence the work reported in this paper.
