# Supplementary material for: A randomized multi-arm open labelled comparative clinical trial report of Pankajakasthuri DiabetEaze powder, a novel polyherbal formulation on the nutritional management and glycemic control in type 2 diabetic and prediabetic patients
Source: Heliyon. 2025 Feb 13;11(4):e42631. doi: 10.1016/j.heliyon.2025.e42631 (PMC11903805; doi:10.1016/j.heliyon.2025.e42631)
Supplement: Multimedia component 1 [file mmc1.docx]

*Statistical analysis*

Descriptive statistics, including frequencies, means, and standard deviations, were employed to characterize the data. Statistical comparisons of primary characteristics and outcomes among the three groups were conducted using generalized linear models, ANOVA, and chi-square tests. An intention-to-treat analysis protocol was applied to analyze data from patients who did not fully adhere to the study protocol. Significance was defined as a p-value less than 0.05. Data analysis was performed using the R software (JAMOVI).
