# Supplementary material for: A randomized multi-arm open labelled comparative clinical trial report of Pankajakasthuri DiabetEaze powder, a novel polyherbal formulation on the nutritional management and glycemic control in type 2 diabetic and prediabetic patients
Source: Heliyon. 2025 Feb 13;11(4):e42631. doi: 10.1016/j.heliyon.2025.e42631 (PMC11903805; doi:10.1016/j.heliyon.2025.e42631)
Supplement: Multimedia component 2 [file mmc2.pdf]

PROTOCOL

PROSPECTIVE RANDOMIZED CLINICAL STUDY TO EVALUATE THE EFFECT OF DIABETEAZE POWDER IN NUTRITIONAL MANAGEMENT OF PRE-DIABETES & TYPE 2 DIABETES MELLITUS.

Protocol Number: PHRF/IEC/001/2021
Version 1:1
Final: 25th March 2021

**Study Sponsor**	**Pankajakasthuri Herbals India Pvt. Ltd, Poovachal Thiruvananthapuram, Kerala, India**

**DR. J. HAREENDRAN NAIR**<br>STUDY DIRECTOR	SIGNATURE…………………
**DR. ARUN PRATAP**<br>PRINCIPAL INVESTIGATOR	**DR.RAJESH P**<br>PRINCIPAL INVESTIGATOR
**DR. RAJANI A NAYAR**<br>CO-INVESTIGATOR	**DR. LEKSHMI R**<br>CO-INVESTIGATOR
**DR. SHAN SASIDHARAN**<br>PRODUCT DEVELOPMENT I/C	

This document contains confidential information regarding above clinical study conducted by Pankajakasthuri Herbal Research Foundation, Poovachal, Thiruvananthapuram-695575, Kerala. Upon acceptance of this document, the investigator and the institution agree to maintain the information as confidential and limit the access to any such information to only those persons who are under, the investigator or the institution direct control and will be engaged in employing such information for the purpose of conducting the study. At no time shall such information available and employed for any other purpose without prior written approval of Pankajakasthuri Herbal Research Foundation. This document is prepared in accordance with the ICH E6 Guidance for Good Clinical Practice.

Independent Ethics Committee of Pankajakasthuri Herbal Research Foundation, Pankajakasthuri Ayurveda Medical College Campus, Kattakkada P.O, Thiruvananthapuram, Kerala, India
EC/NEW/INST/2020/1089

PROTOCOL SYNOPSIS

NAME AND ADDRESS OF PRINCIPAL INVESTIGATORS

1. Dr. Arun Pratap,
   Professor,
   Department of Kayachikitsa,
   Pankajakasthuri Ayurveda Medical College & PG Centre,
   Killy, Kattakada, Thiruvananthapuram, Kerala
2. Dr. Rajesh P,
   Senior Consultant in Medicine & Diabetology,
   Neyyar Medicity,
   Killy, Kattakada, Thiruvananthapuram, Kerala

NAME AND ADDRESS OF CO- INVESTIGATORS

1. Dr. Rajani A Nayar,
   Vice Principal & Professor,
   Department of Agadatantra,
   Pankajakasthuri Ayurveda Medical College & PG Centre,
   Killy, Kattakada, Thiruvananthapuram, Kerala
2. Dr. Lekshmi R
   Medical Superintendent & Assistant Professor,
   Department of Kayachikitsa,
   Pankajakasthuri Ayurveda Medical College & PG Centre,
   Killy, Kattakada, Thiruvananthapuram, Kerala

MONITORING TEAM

1. Dr. Sreejith Satheesan,
   Associate Professor,
   Department of Salyatantra,
   Pankajakasthuri Ayurveda Medical College & PG Centre,
   Killy, Kattakada, Thiruvananthapuram, Kerala
2. Dr. Arjun Chand C.P,
   Associate Professor,
   Department of Kayachikitsa,
   Pankajakasthuri Ayurveda Medical College & PG Centre,
   Killy, Kattakada, Thiruvananthapuram, Kerala

3. Dr. Sajna S.A,
   Associate Professor,
   Department of Rasasastra & Bhaishajyakalpana,
   Pankajakasthuri Ayurveda Medical College & PG Centre,
   Killy, Kattakada, Thiruvananthapuram, Kerala
4. Dr. Sandhu G. Joseph,
   Assistant Professor,
   Department of Dravya Guna Vijnanam,
   Pankajakasthuri Ayurveda Medical College & PG Centre,
   Killy, Kattakada, Thiruvananthapuram, Kerala

SPONSOR

Pankajakasthuri HerbalsIndiaPvt. Ltd, Thiruvananthapuram, Kerala, India

TRIAL MONITOR

Dr Shan Sasidharan
Head, Clinical Research & Director, R&D Department,
Pankajakasthuri Herbal Research Foundation
Pankajakasthuri Ayurveda Medical CollegeCampus,
Kattakada P.O, Thiruvananthapuram

OTHER EMERGENCY TELEPHONE NUMBERS

1. Dr. Arun Pratap - +91 9567256333
2. Dr.Rajesh P - +919447034039
3. Dr. Rajani A Nayar - +919447587082
4. Dr. Lekshmi R -+91 8075735501
5. Dr. Sreejith Satheesan - +919447791353
6. Dr. Arjun Chand C.P - +918072948368
7. Dr. Sajna S.A - +919746123343
8. Dr. Sandhu G. Joseph - +91 9482331617

IEC & IEC APPROVAL NUMBER

Independent Ethics Committee of Pankajakasthuri Herbal Research Foundation,Pankajakasthuri Ayurveda Medical College Campus, Kattakkada P.O,Thiruvananthapuram, Kerala, India.

IEC Approval Number:

CLINICAL TRIAL SUMMARY

**TITLE (GENERAL)**	Prospective Randomized Clinical Study to Evaluate the Effect of DiabetEaze Powder in Nutritional Management of Pre-Diabetes & Type 2Diabetes Mellitus.
**TITLE (SCIENTIFIC)**	Open, Realistic, Prospective, Randomized, Controlled, Interventional Clinical Study of DiabetEaze Powder in Nutritional Management of Pre-Diabetes & Type 2 Diabetes Mellitus.
**INVESTIGATOR/ TRIALLOCATION**	Pankajakasthuri Ayurveda Medical College & PG Centre, Killy, Kattakada, Kerala and Neyyar Medicity, Kattakada, Trivandrum
**STUDY OBJECTIVES**	**Primary objectives:**<br>❖ Clinical Nutritional Assessment of DiabetEaze Powder in Type 2 Diabetes Mellitus.<br>❖ To assess the variation in Quality of Life in terms of direct symptoms.<br>❖ To assess the role of DiabetEaze Powder in managing nutritional inadequacy in Type 2 Diabetes Mellitus.<br>**Secondary objectives:**<br>❖ Assessment of hypoglycaemic effect of DiabetEaze Powder.<br>❖ Assessment of effect of DiabetEaze Powder in Pre-Diabetes.<br>❖ Assessment of reduction in complications.
**STUDY DESIGN**	Open, Realistic, Prospective, Randomized, Controlled, Interventional Clinical Study.
**STUDY POPULATION**	Subjects from OPD of Pankajakasthuri Ayurveda Medical College Hospital, Kattakada, Trivandrum and Neyyar Medicity, Kattakada, Trivandrum, satisfying the inclusion criteria.

**MAIN INCLUSION CRITERIA**	1. Willing to sign consent form<br>2. Men and Women aged 40 years or greater and not older than 65 years.<br>3. Subjects with clinical evidence of Type 2 Diabetes Mellitus.<br>4. Subjects of Pre-Diabetes with HbA1c range between 5.7% and 6.4% and/or OGTT range between 140 mg/dl and 199 mg/dl.
**MAIN EXCLUSION CRITERIA**	1. Presence of conditions like Medical, Psychological, Social or Alcohol abuse that would interfere with participation in the trial.<br>2. Subjects with known allergy to any of the ingredients of the drug product.<br>3. Pregnant or Lactating women.<br>4. Subjects with Type1 Diabetes Mellitus.<br>5. Subjects undergoing Insulin therapy.<br>6. Other conditions like CKD, Malignancies etc.<br>7. Post-surgical cases mainly GI diseases.<br>8. Patients under Steroid intake.
**EXPECTED NUMBER OF SUBJECTS**	180
**NAME OF INVESTIGATIONAL PRODUCT**	DiabetEaze Powder
**FORMULATION**	Ingredients of DiabetEaze Powder is as follows:<br>*Curcuma longa Linn* (Haridra)<br>*Emblica officinalis Gaertn*(Amalaki)<br>*Strychnos potatorum Linn* (Kataka)<br>*Salacia reticulata Linn* (Ekanayakam)<br>*Carum carvi Linn*(Krishna Jeeraka)<br>*Mimosa pudica* (Lajjalu)<br>*Moringa oleifera* (Shigru)<br>*Tinospora cordifolia* (Guduchi)

	Andrographis paniculata(Kalamegha)<br>*Artocarpus heterophyllus*(Panasa)<br>*Eleusine coracana*(Ragi)<br>*Hordeum vulgare* (Yava)<br>*Anacardium occidentale*(Vrikkaphala)<br>*Theobroma cacao* (Rasalaphala)
**ROUTE OF ADMINISTRATION**	Oral
**PROOF OF CONCEPT**	This formula of the nutritional supplement is based on traditional knowledge.
**INVESTIGATIONAL PRODUCT, MANUFACTURING, AND DOSAGE**	The study drug DiabetEaze is a poly herbal formulation for providing nutritional support to Pre-Diabetes & Type 2 Diabetes Mellitus. Also, it is aimed to reduce complications and to improve quality of life in Type 2 Diabetes Mellitus. The study drug will be manufactured in GMP certified factory of Pankajakasthuri Herbals India Pvt. Ltd, Thiruvananthapuram, Kerala, India.<br>Dosage– 10g/day [5g twice daily]
**EVALUATION CRITERIA**	Clinical, Haematological and Biochemical assessments
**ASSESSMENT SCHEDULE**	The initial assessment will be done before the commencement of the study (0th day) and simultaneously the study will be initiated. Further assessment will be done on the 3rd month and will be followed on every six months till the completion of the study (i.e. 90th, 180th, 365th, 545th& 730th day). Interim data analysis will be done at the end of first six months (180th day) and report will be published in reputed journal.
**STATISTICAL CONSIDERATION**	Appropriate statistical analysis will be performed. *p value* ≤0.05 indicates that the study is statistically significant.
**DURATION OF STUDY**	The initial study period is 24 months, if required the study can be extended.
**REFERENCE THERAPY**	Placebo and Standard of Care (SOC) will be given to control groups. In the study groups the trial drug and SOC will be

	given. Placebo for this trial will be given in the similar form as that of the trial drug. Placebo must be consumed according to the same procedures and guidelines as DiabetEaze Powder.
**CRITERIA FOR EVALUATION:**	**Efficacy**:<br>• Fasting Blood Sugar<br>• Post Prandial Blood Sugar<br>• Oral Glucose Tolerance Test<br>• HbA1c<br>• Urine Sugar<br>• Investigation for Vitamins & Minerals<br>• Investigations for Diabetic Nephropathy<br>• Investigations for Diabetic Cardiopathy<br>• Investigations for Diabetic Neuropathy<br>• Investigations for Diabetic Retinopathy<br>• Other Laboratory investigations– CBC, Lipid Profile, LFT, RFT, CRP, Urine Examination etc.<br>**Safety**:<br>• ADR/AEs using MedDRA<br>• RFT & LFT

PROTOCOL

PROSPECTIVE RANDOMIZED CLINICAL STUDY TO EVALUATE THE EFFECT OF DIABETEAZE POWDER IN NUTRITIONAL MANAGEMENT OF PRE-DIABETES & TYPE 2 DIABETES MELLITUS

Version 1:1 Dated 25thMARCH 2021

PROTOCOL APPROVAL

The signature of the Principal Investigators constitutes an agreement that this study will be conducted according to all stipulations, clinically and administratively, as stated in the protocol, including all statements as to confidentiality. It is agreed that the conduct and results of this study will be kept confidential and that the case report forms and other pertinent data will become the property of Pankajakasthuri Herbal Research Foundation, Thiruvananthapuram. It is agreed that the protocol contains all necessary information required to conduct the study as outlined in the protocol, and that the study will not be initiated without the approval of an appropriate Institutional Review Board or Ethics Review Committee. It is agreed that all participants in this study will provide written informed consent in accordance with ICH Guidelines for Good Clinical Practice and the requirements specified in the Code of Federal Regulations (21 CFR Parts 50, 56, 312) and/or the Declaration of Helsinki. All participants will also be informed that their medical records will be kept confidential except for review by authorized representatives of Pankajakasthuri Herbal Research Foundation and its associates, the DCGI, ICMR or other regulatory agencies.

Dr. ARUN PRATAP

1. PRINCIPAL INVESTIGATOR	SIGNATURE	DATE

Dr. RAJESH P

2. PRINCIPAL INVESTIGATOR	SIGNATURE	DATE

By signing the above,we agree to perform the study in accordance with the protocol, ICH Good Clinical Practice (GCP) guidelines, and all applicable regulations.

BACK GROUND

Diabetes Mellitus (DM) is a chronic metabolic disorder characterized by hyperglycaemia with or without glycosuria, resulting from absolute or relative deficiency of insulin, its action or both[1]. Latest studies show, Diabetes is the fastest growing health emergencies of the 21st century. 'Pre-Diabetes' is a term increasingly used for people with impaired glucose tolerance and/or impaired fasting glucose. It signifies a risk of future development of type 2 DM and Diabetes-related complications [2]. According to the International Diabetes Federation, India is one among the epicentre of DM and it was found that in 2000, the global estimate of adults living with Diabetes was 151 million. Also, IDF estimates that there will be 578 million adults with Diabetes by 2030, and 700 million by 2045[3, 4].In course of time, individuals with Diabetes are more susceptible to develop any associated complications of kidneys, heart, blood vessels, nerves and eyes. As the number of diabetic patients has been increasing in India, diabetic complications are the most important target to be prevented.

RATIONALE

Indian Council of Medical Research initiated a multicenter study on DM on 1971. The prevalence of the disease was found to be 2.1% in the urban areas and 1.5% in the rural areasin six cities and surrounding villages in India including Ahmadabad, Kolkata, Cuttack, Delhi, Pune and Trivandrum [5]. Prevalence of Diabetes in India study, evaluated the prevalence of Diabetes mellitus in small towns and villages of India, which was found to be 5.9% and 2.7%, respectively [6].

Once Diabetes is established, it tends to be lifelong. Prognosis depends largely on the presence or absence of chronic vascular complications. Present day treatment has served to prevent or successfully overcome acute complications like ketoacidosis, infections, hypoglycaemia and lactic acidosis. There are long term complications affecting the heart, kidneys, eyes, nerves and blood vessels. These complications can be prevented to a great extent by rigid diet control, exercise and regular therapy. Once established the degenerative complications progress relentlessly and lead to damage of vital organs like the kidneys, heart and brain unless properly managed [7]. For people living with Diabetes, access to affordable treatment, including insulin, is critical to their survival. There is a globally agreed target to halt the rise in Diabetes and obesity by 2025[8].

JUSTIFICATION FOR CONDUCTING THE TRIAL

The Dietary Supplement Health and Education Act (DSHEA) of 1994 define a dietary supplement as a "product taken by mouth that contains a dietary ingredient intended to supplement the diet." These dietary ingredients may consist of "vitamins, minerals, herbs, amino acids, and other botanicals and substances such as enzymes, organ tissues, glandulars, and metabolites" [9]. According to the 2017 Standards of Medical Care in Diabetes, "Each person with diabetes should be actively engaged in education, self-management, and treatment planning with his or her health care team, including the collaborative development of an individualized eating plan" [10]. Thus nutritional therapy is much essential for Pre-diabetic and Diabetic patients for providing optimum diabetes care.

Studies have shown that poor eating habits are the main factor behind the disease DM. Also, diabetic subjects usually follow unhealthy eating habits which impair daily health and wellbeing. This habit can also affect the mental health, energy levels, complexion, and overall well-being. More over it is scientifically proven that DM subjects have decreased levels of Vitamin A, C and E. To overcome this, a supplement can be used to fill in nutritional gaps of the diet. Conventional medical system had a great deal to offer in this regard. Various ingredients in DiabetEaze Powder such as Amalaki, Krishna Jeeraka, Shigru, Kalamegha, Ragi and Yava provides minerals, micro elements and amino acids which are essential for a diabetic person to overcome the oxidative stress. Also Vitamin B complex, C and E in Amalaki, Krishna Jeeraka, Shigru, Kalamegha, Ragi, Yava, Vrikkaphala and Panasa will help to overcome oxidative stress, boost the immune system and reduces the risk of development of various diabetic complications. DiabetEaze powder also ensures that person is getting a measurable amount of essential nutrients which make up for the poor nutrient content of the many foods one eats.

STUDY OBJECTIVES

Primary objective:

- Clinical Nutritional Assessment of DiabetEaze Powder in Type 2 DM
- To assess the variation in Quality of Life in terms of direct symptoms.
- To assess the role of DiabetEaze Powder in managing nutritional inadequacy in DM.

Secondary objectives:

- Assessment of hypoglycaemic effect of DiabetEaze Powder.
- Assessment of effect of DiabetEaze Powder in Pre-Diabetes.
- Assessment of reduction in complications.

ELIGIBILITY CRITERIA

Subjects are eligible to be included in the study, if they meet the following criteria:

Main inclusion criteria

1. Willing to sign consent form
2. Men and women aged 40 years or greater and not older than 65 yrs.
3. Subjects with clinical evidence of Type 2 DM
4. Subjects of Pre-Diabetes with HbA1c range between 5.7% and 6.4% and/or OGTT range between 140 mg/dl and 199 mg/dl.

Main exclusion criteria

1. Presence of conditions like medical, psychological, social or alcohol abuse that would either interfere with participation in the trial.
2. Subjects with known allergy to any of the ingredients of the drug product.
3. Pregnant or lactating women
4. Subjects with Type1 DM.
5. Subjects undergoing insulin therapy.
6. Subjects with fluctuating blood sugar level.
7. Other conditions like CKD, malignancies etc.
8. Post-surgical cases mainly GI diseases.
9. Patients under steroid intake

DURATION OF THE STUDY

The initial study period is 24 months. If required, the study can be extended.

STUDY PERIOD

Begins at the first trial drug dose and ends at the trial completion. These dates are reported on the both paper based and electronic version Case Report Form (CRF/eCRF).

STUDY COMPLETION AND END OF THE STUDY

This trial will be considered complete (i.e. the scientific evaluation will be complete) following the evaluation of final outcome data. Investigators will continue the study if required. The End of the trial refers to the date of last visit (Day 730) or last schedule assessment for the last subject. The end of trial occurs after the study completion and after the last subject has discontinued the trial.

METHODOLOGY

Sample size

180

Type of study design

Open, realistic, prospective, randomized, controlled, interventional clinical study.

Participant recruitment process

Subjects must meet the inclusion criteria and have none of the exclusion criteria in order to be eligible for randomization in this study. Subjects who meet all eligibility criteria, and have signed informed consent will be included under Baseline study visit. Once the subject is randomized the Sponsor Representative or the Study Investigator will provide the study staff with a unique randomization number for the subject. At the Randomisation, subjects will be centrally, dynamically randomised in a 1:1:1:1:1:1 fashion to each of the following six groups, namely two controls and four trial groups each with 30 subjects.

1. Group 1: Control group for Pre-Diabetes (30 subjects).
2. Group 2: Study group for Pre-Diabetes (30 subjects).
3. Group 3: Control group for Type II DM (30 subjects).
4. Group 4: Study group for Type II DM – Study drug will be administered as add on therapy with Allopathic medicines (30 subjects).
5. Group 5: Study group for Type II DM – Study drug will be administered as add on therapy with AYUSH medicines (30 subjects).
6. Group 6: Study group for Type II DM – Study drug will be administered along with life style management (30 subjects).

Conduction of trial and Assessments

Description of type- Design of the trial to be conducted

The trial is planned as Open, Realistic, Prospective, Randomized, Controlled, Interventional Clinical study. The study will be conducted in six groups of which two groups will be control and rest four will be trial groups. The initial assessment will be done before the commencement of the study (Baseline/0th day) and simultaneously the study will be initiated. Further assessment will be done on the 3rd month and will be followed on every six months till the completion of the study (i.e. 90th, 180th, 365, 545 & 730th day). Interim data analysis will be done at the end of first six months (180th day) and report will be published.

Laboratory Samples

Hospital laboratory can be used to access the following tests. If the hospital laboratory has limitation an outside laboratory with NABL accreditations shall be arranged for sample analysis. The following investigations will be conducted:

- **FASTING BLOOD SUGAR**
- **POST PRANDIAL BLOOD SUGAR**
- **ORALGLUCOSE TOLERANCE TEST**
- **HbA1C**
- **URINE SUGAR**
- **INVESTIGATION FORVITAMINS & MINERALS**
- **INVESTIGATIONS FOR DIABETIC NEPHROPATHY**
- **INVESTIGATIONS OF DIABETIC CARDIOPATHY**
- **OTHER TESTING METHOD**S **- CBC, LIPID PROFILE, LFT, RFT, CRP, URINE EXAMINATION etc.**

Data Collection

In addition to the CRFs, individual subject files or subject medical records as well as Sponsor approved source data information will be maintained. These files constitute source data, and it is required that they be signed or initialled and dated by the staff recording the data. Data will be entered into CRFs. The CRFs will be kept current, so that they reflect the latest observations on the subjects enrolled in the study. In this trial CRFs designed will be both paper based and electronic version. The CRFs will be reviewed to verify changes made, reason for change, date of change, and person making changes.

The original signed informed consent form will be available for review at each study visit. All records will be kept in accordance with applicable national laws and regulations. Analytical data from the laboratory will be received and filed along with source documents.

Informed Consent

Fully informed consent will be obtained before any study specific procedures are performed. The content and process of obtaining informed consent must be in accordance with all applicable ethical and regulatory requirements. Informed consent form must be signed by the subject voluntarily. In a situation where a subject is unable to provide consent for him/her, informed consent will be obtained from a Legally Authorized Representative (LAR) / next of kin / legally appointed individual in person in the presence of an impartial witness.

Screening / Randomization, Visit – Day 0

Subjects must meet the inclusion criteria and have none of the exclusion criteria in order to be eligible for randomization in this study. Subjects who meet all eligibility criteria and have signed informed consent will be included under Baseline study visit. Randomization minimizes systematic bias in the selection and assignment of patients to study therapy and provides justification for inferential statistical methods to be used on data from this study. Once the subject is randomized the sponsor representative or the study Investigator will provide the study staff with a unique randomization number for the subject.

Following assessments must be made at this Baseline visit for each study subject:

- Demographics
- Medical History (all known information regarding the subject's health history/relevant surgeries/interventions, prior to signing the informed consent).
- Vital signs: Body temperature, Pulse – beats per minute (BPM), Blood Pressure (BP) mmHg, Respiratory Rate – breaths per minute
- Concomitant medications
- General Examination -
- Systemic Examination: Examination of Respiratory, Cardiovascular, Nervous, Musculo-skeletal and Integumentary System
- BMI
- Blood will be obtained for following laboratory tests:

• FBS	• HBA1C	• Urine- sugar
• PPBS	• OGTT	

- Investigation for Vitamins & Minerals, Diabetic Nephropathy, Diabetic Cardiopathy and other investigations like CBC, Lipid Profile, RFT, LFT, CRP, Urine Examinations etc.
- Screening for Diabetic Neuropathy& Retinopathy.

Short term assessment, Visit – Day 90

Following assessments must be made at this Baseline visit for each study subject:

- Vital signs: Body temperature, Pulse – beats per minute (BPM), Blood Pressure (BP) mmHg, Respirations – breaths per minute
- Concomitant medications
- General Examination

- Systemic Examination: Examination of Respiratory, Cardiovascular, Nervous, Musculo-skeletal and Integumentary System
- BMI
- Blood will be obtained for following laboratory tests:
- FBS
- HBA1C
- Urine- sugar
- PPBS
- OGTT
- Investigation for Vitamins & Minerals, Diabetic Nephropathy, Diabetic Cardiopathy and other investigations like CBC, Lipid Profile, RFT, LFT, CRP, Urine Examinations etc.
- Screening for Diabetic Neuropathy & Retinopathy.

Interim assessment, visit – Day 180

Following assessments must be made at this Baseline visit for each study subject:

- Vital signs: Body temperature, Pulse – beats per minute (BPM), Blood Pressure (BP) mmHg, Respirations – breaths per minute
- Concomitant medications
- General Examination
- Systemic Examination: Examination of Respiratory, Cardiovascular, Nervous, Musculo-skeletal and Integumentary System
- BMI
- Blood will be obtained for following laboratory tests:
- FBS
- HBA1C
- Urine- sugar
- PPBS
- OGTT
- Investigation for Vitamins & Minerals, Diabetic Nephropathy, Diabetic Cardiopathy and other investigations like CBC, Lipid Profile, RFT, LFT, CRP, Urine Examinations etc.
- Screening for Diabetic Neuropathy & Retinopathy.

End of treatment/Trial visit (EOT) - Day 730

Following assessments must be made at this EOT visit for each study subject

- Vital signs: Body temperature, Pulse – beats per minute (BPM), Blood Pressure (BP) mmHg, Respirations – breaths per minute
- Concomitant medications
- General Examination

- Systemic Examination: Examination of Respiratory, Cardiovascular, Nervous, Musculo-skeletal and Integumentary System
- BMI
- Blood will be obtained for following laboratory tests:
  - FBS
  - PPBS
  - HBA1C
  - OGTT
  - Urine- sugar
- Investigation for Vitamins & Minerals, Diabetic Nephropathy, Diabetic Cardiopathy and other investigations like CBC, Lipid Profile, RFT, LFT, CRP, Urine Examinations etc.
- Screening for Diabetic Neuropathy & Retinopathy.

Assessment of effect of trial drug: Day 01 to EOS

The initial assessment will be done before the commencement of the study (0th day) and simultaneously the study will be initiated. Further assessment will be done on the 3rd month and will be followed on every six months till the completion of the study (i.e. 90th, 180th, 365, 545 & 730^{th}day). Interim data analysis will be done at the end of first six months (180th day) and report will be published.

Flow chart of the proposed study

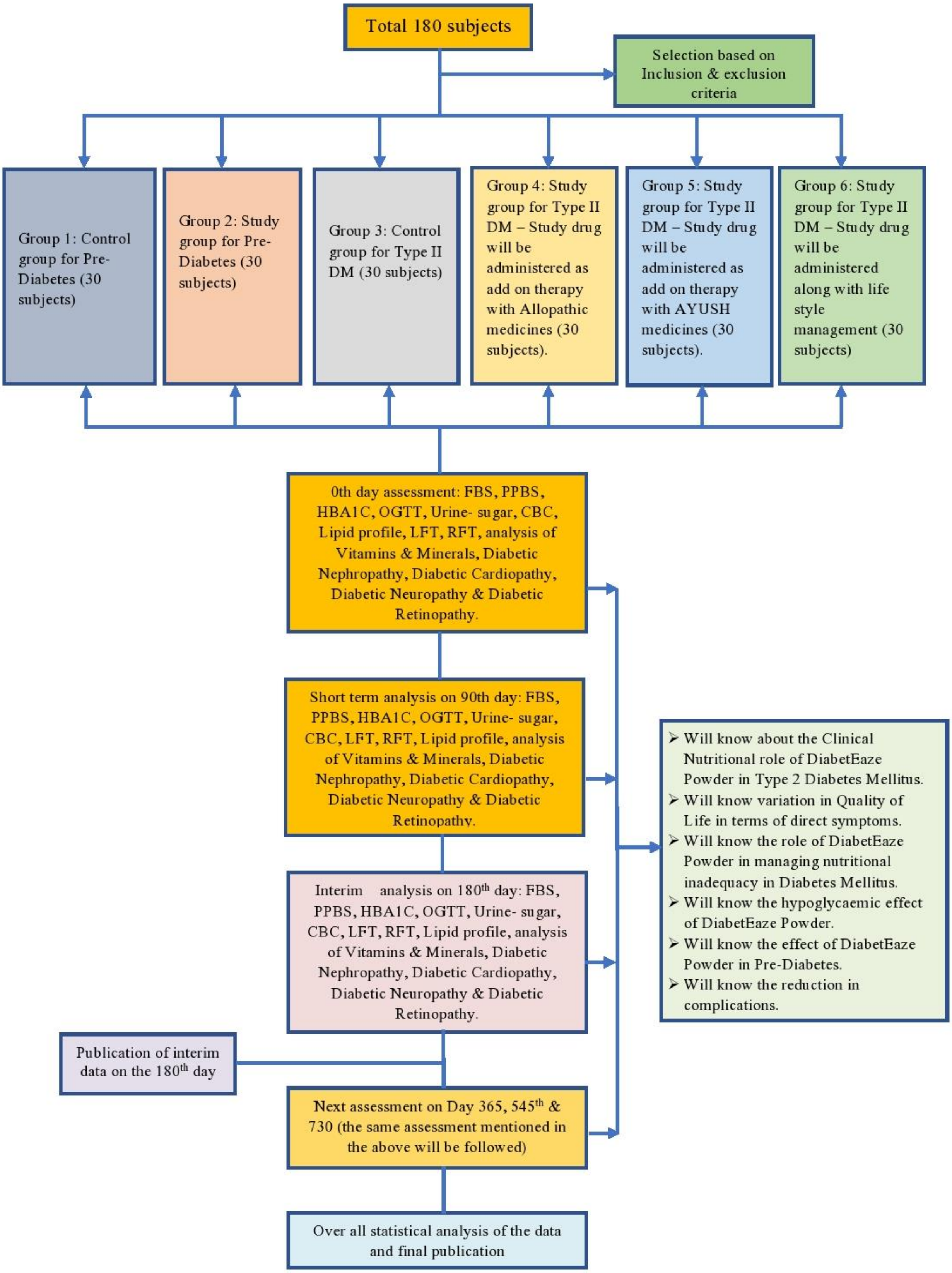

Major milestones of the proposed study

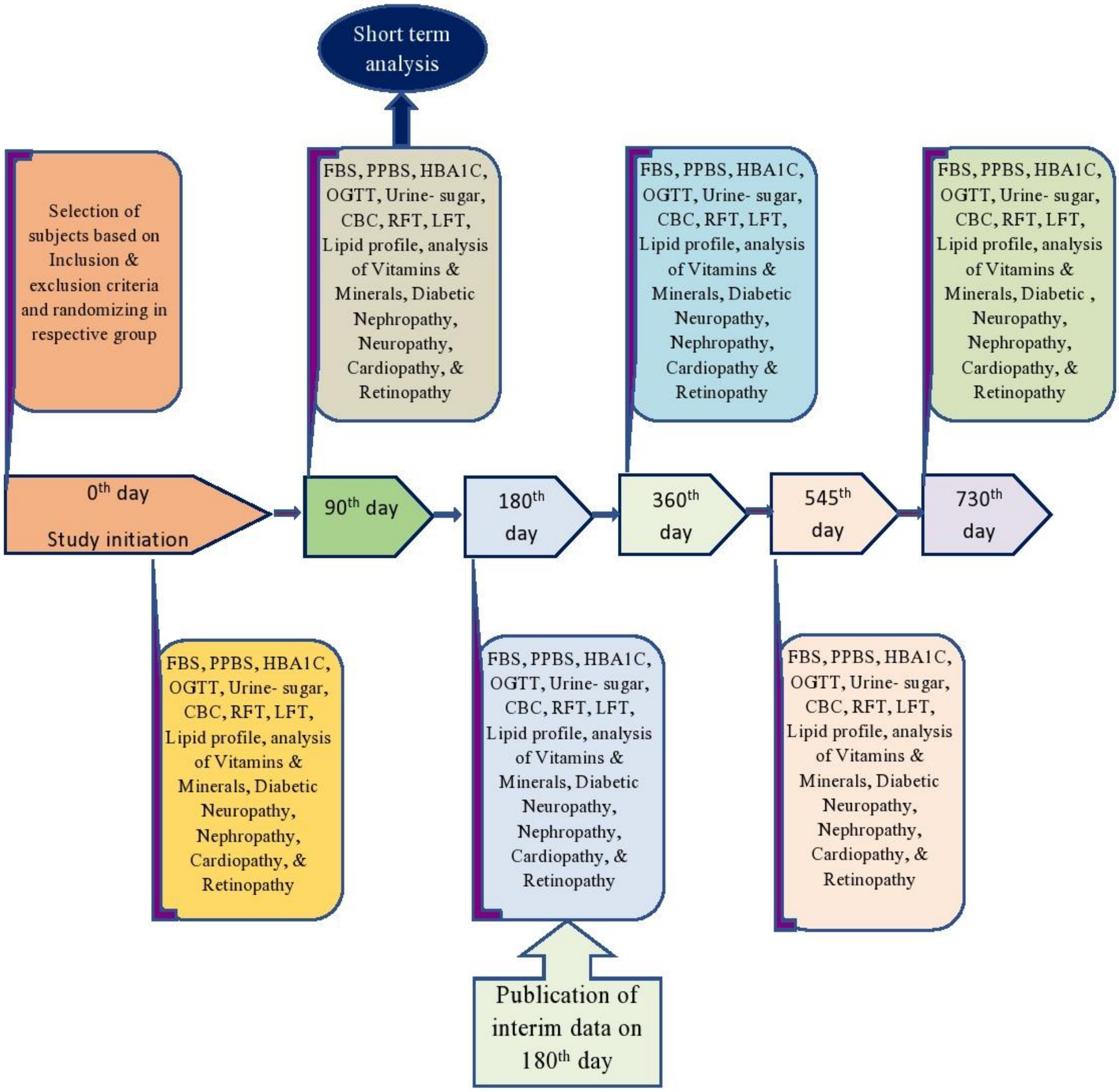

PLAN OF STATISTICAL ANALYSIS

Appropriate statistical tools will be used for the study.

Criteria for Discontinuation from Study Drug or Study Participation

Withdrawal from receiving study drug

A subject may remain in the study but discontinue the administration of study drug at any time during treatment. Reasons for this may include:

- A subject's desire to withdraw for any reason.
- Intolerable adverse event(s)
- Subject's request
- A subject's substantial non-compliance (e.g. visits non-compliance) after agreement with the Sponsor.
- Investigator's judgment (e.g. related AE)

In any above case of withdrawal or lost to follow-up, the subject's status shall be documented appropriately in the subject source document and will be reported to Sponsor.

Safety Assessments and Reporting

Adverse Events

An Adverse Event (AE) is defined as any untoward medical occurrence in a subject administered with a pharmaceutical product, which does not necessarily have a causal relationship with this treatment. An AE can therefore, be any unfavourable and unintended sign, symptom, or disease temporarily associated with the use of a medicinal (investigational) product, whether or not considered related to the medicinal (investigational) product. Examples of adverse events include:

- Increase in severity of a baseline event
- Clinically significant worsening of lab values post baseline
- A new event with an onset date post Baseline

As far as possible, each AE will also be described by:

- The duration (start and end dates)
- The severity grade (Grade 1/mild, Grade 2/moderate, Grade 3/severe, Grade 4/Potentially life threatening as defined by the DAIDS Toxicity Table)
- The relationship to the study drug (none, unlikely, related)
- Whether the AE involved bleeding of any kind
- The action(s) taken
- The outcome

Serious Adverse Events

Information about all Serious Adverse Events (SAEs) will be collected and recorded on the SAE Report form within 24 hours of learning of its occurrence. A paper SAE form should be completed by the investigators and faxed or emailed to the Sponsor within 24 hours using the safety reporting contact information provided. An SAE is any undesirable sign, symptom, or medical condition which:

- Is fatal or life-threatening (i.e. an event with an outcome of 'Death'), or
- Requires or prolongs inpatient hospitalization, or
- Results in persistent or significant disability/incapacity, or
- Is medically significant, as determined by a qualified health professional, may jeopardize the subject, and may require medical or surgical intervention to prevent one of the outcomes listed above
- Any adverse event causing the need for unblinding will be handled as a serious adverse event.

Specification of safety parameters

For ensuring safety of the subjects, the review on90th, 180th, 365, 545 & 730th day is scheduled. If found necessary, short-term assessments other than the above stated will be performed. However, in case of emergency conditions between treatments, the trial drug will be discontinued and conditions will be managed accordingly.

Provision of auxiliary care

In the event of a medical emergency, where it is imperative to know which treatment arm the subject was randomized to in order to make future treatment decisions, the Investigator make necessary arrangement for expert management.

Medical Monitor Coverage

Each subject will be provided with contact information of the Principal Investigators. When a subject present to a medical facility where the treating physician or health care provider requires access to a physician who has knowledge of the investigational product and the clinical study protocol and the Principal Investigator is not available, the treating physician or health care provider can contact the Trial Monitor.

Changes to the Protocol and Informed Consent Form

Study procedures will not be changed without the mutual agreement of the Investigators and Sponsor. If there are any substantial changes to the study protocol, then these changes will be documented in a study protocol amendment and if required, in a new version of the study protocol. The amendment is to be approved by the relevant IRB/IEC and if applicable, also

the National Regulatory Authority approval, before implementation. Local requirements are to be followed for revised protocols.

DATA MANAGEMENT

Documentation

Case report forms (CRFs) will identify each subject-by-subject number and subject's initials (per local regulatory guidelines). Originals or copies of all source documents, and correspondence will be kept on file at the investigational site.

The data required by the protocol will be recorded in the appropriate pages of CRFs. All source data will be available to the study monitor who may perform a 100% data check (comparison of the data recorded in the eCRF with those in the source documents). The source data will also be available for an audit by the Sponsor and all applicable regulatory agencies at any time.

Subject Data Protection

The Informed Consent Form (ICF) will incorporate (or, in some cases, be accompanied by a separate document incorporating) wording that complies with relevant data protection and privacy legislation.

Storage and maintenance of data

The Investigator follows the principles outlined in the Clinical Study Agreement on the archival of trial related documents post study. Local IRB procedures and country regulations needs to be followed on archival of documents.

Ethics &Good Clinical Practice

This trial will be carried out in compliance with the protocol and in accordance with all applicable Standard Operating Procedures (SOPs). These will be designed to ensure adherence to GCP, as described in:

ICH Harmonized Tripartite Guidelines for Good Clinical Practice 1996. The Investigator agrees, when signing the protocol, to adhere to the instructions and procedures described in it, and thereby to adhere to the principles of GCP to which it conforms.

Institutional Review Board/Independent Ethics Committee

Before implementing this study, the protocol, the proposed informed consent form and other information to subjects will be reviewed by a properly constituted IEC. A signed and dated statement that the protocol and informed consent have been approved by the IEC will be given to the Sponsor before study initiation. The name and occupation of the Chairman and

the members of the IEC will be supplied to the Sponsor. This committee will approve any amendments to the protocol, other than administrative changes.

Audits and Inspections

Authorized representatives of Sponsor, a regulatory authority, or an IRB/IEC may perform audits or inspections at the centre, including source data verification. The purpose of an audit or inspection is to systematically and independently examine all study-related activities and documents, to determine whether these activities were conducted, and data were recorded, analyzed, and accurately reported according to the protocol, GCP, guidelines of the ICH, and any applicable regulatory requirements. The Investigator will contact Sponsor immediately if contacted by a regulatory agency about an inspection at the site.

Publication of Results

Any formal presentation or publication of data from this study will require prior approval by the Sponsor, and may be considered as a joint publication by the Investigators and the Sponsor. Primary authorship may be determined by the Sponsor. Publication of subsets of data will be subject to review by the Sponsor. All data remain the property of the Sponsor, otherwise the legal authorities except, the copies maintained in compliance with ICH GCP and other regulations.

References

1. K.V.Krishna Das. Text Book of medicine, 6th Edition, Jaypee Brothers Medical Publishers (P) Ltd, New-Delhi; 2017, Pg:577.
2. International Diabetes Federation: IDF Diabetes Atlas (2019) http://www.idf.org/idf-diabetes-atlas-nineth-edition. Accessed March 2020.
3. International Diabetes Federation: IDF Diabetes Atlas (2019) http://www.idf.org/idf-diabetes-atlas-eight-edition. Accessed March 2020.
4. International Diabetes Federation. [online], IDF Diabetes Atlas 9th edition 2019
5. Ahuja, M. M. S. (ed.) in Epidemiological Studies on DMin India in Epidemiology of Diabetes in Developing Countries 29–38 (Interprint, 1979).
6. https://www.nature.com/articles/nrendo.2016.53
7. K.V.Krishna Das. Text Book of medicine, 6th Edition, Jaypee Brothers Medical Publishers (P) Ltd, New-Delhi; 2017, Pg:599.
8. www.who.int › Health topics:https://www.who.int/news-room/fact-sheets/detail/diabetes.
9. https://clinical.diabetesjournals.org/content/28/1/35.Assessed March 2020
10. Evert AB, Boucher JL, Cypress M, et al. Nutrition therapy recommendations for the management of adults with diabetes. Diabetes Care. 2014;37 Suppl 1:S120-143.

APPENDIX 1 / അപ്പൻഡിക്സ് – 1

CONSUMER INFORMATION SHEET/ഉപഭോക്തൃ വിവരപ്പട്ടിക

NAME OF THE PROJECT/പ്രോജക്ടിന്റെ പേര്:

A RANDOMIZED STUDY TO EVALUATE THE EFFECT OF DIABETEAZE POWDER IN NUTRITIVE MANAGEMENT OF TYPE II DIABETES MELLITUS

You are being invited to take part in a research study. Before you decide, it is important for you to please take time to read the following instructions carefully and discuss the same with your friends or relatives and if you would like to get more information you may consult the team of researchers undertaking the project and you can take time to decide whether or not to to take part in the study.

ഈ പ്രോജക്ടിന്റെ പഠനാവശ്യങ്ങൾക്കായി താങ്കളെ ക്ഷണിച്ചുകൊള്ളുന്നു. താങ്കൾക്ക് ഇതിന്റെ ഭാഗമാകുവാൻ താല്പര്യമു ങ്കിൽ ചുവടെ ചേർത്തിരിക്കുന്ന നിയമാവലികൾ ശ്രദ്ധയോടെ സമയമെടുത്ത് വായിച്ച് മനസിലാക്കിയ ശേഷം നിങ്ങളുടെ സുഹൃത്തുക്കൾ, ബന്ധുക്കൾ എന്നിവരോട് ചർച്ച ചെയ്യാവുന്നതാണ്. ഇതിനെ സംബന്ധിച്ച് എന്തെങ്കിലും ആശങ്കയു ങ്കിൽ ഗവേഷണ സംഘവുമായി ചർച്ച ചെയ്ത് തീരുമാനം അറിയിക്കാവുന്നതാണ്.

Q 1) What is the purpose of the study?

ചോദ്യം 1) ഈ പഠനത്തിന്റെ ആവശ്യകത എന്ത്?

Ans: Consumers of "**DIABETEAZE**" who have participated in the in house pilot study have reported that the product is effective for various ailments like tiredness, pallor, increased hunger, increased thirst, dryness of throat etc associated with Diabetes. It has also proven effectiveness on glycaemic levels of diabetes patients. It is also proven to have wonderful result as a nutritive supplement in diabetic patients. This study is designed as a scientific method to prove or disprove the various claims about the product and to find its medicinal and nutritive effectiveness.

ഉത്തരം: "ഡയബറ്റീസ്" എന്ന ഉല്പന്നം സേവിച്ച ഉപഭോക്താക്കൾ അവരിൽ ഡയബറ്റീസ് മൂലം ഉ ായ ആരോഗ്യപ്രശ്നങ്ങളായ ക്ഷീണം, വിളർച്ച, ദാഹം, അമിതമായ വിശപ്പ്, തൊ വരൾച്ച തുടങ്ങിയവയ്ക്ക് കാര്യമായ മാറ്റങ്ങൾ വന്നതായി സൂചിപ്പിച്ചിട്ടു ്. ഡയബറ്റീസ് രോഗികളിൽ രക്തത്തിലെ ഷുഗർന്റെ അളവിലും കാര്യമായ മാറ്റം വരുത്തുവാൻ സാധിച്ചതായി ക ത്തിയിട്ടു ്.

ഡയബറ്റീസ് രോഗബാധിതരിൽ ഇതൊരു ഉത്തമ പോഷക ചേരുവ ആയും തെളിയിക്കപ്പെട്ടിട്ടു ്. അതിനാൽ ഈ ഉല്പന്നത്തിന്റെ യുക്തിപൂർവ്വമായ ഉപയോഗം കൊ ് പ്രമേഹ ബാധിതരുടെ ആരോഗ്യപ്രശ്നങ്ങൾക്ക് കാതലായ മാറ്റം വരുത്തുവാൻ സാധിക്കുന്നു.

ഈ വസ്തുതകൾ ശാസ്ത്രീയമായി തെളിയിക്കുവാനും ഈ ഉല്പന്നത്തിന്റെ പോഷക ആരോഗ്യ ഔഷധ മൂല്യങ്ങൾ ക ത്തുന്നതിനും വേ ിയാണ് ഈ പഠനം നടത്തുന്നത്.

Q 2) Why have I been chosen?

ചോദ്യം 2) എന്തു കൊ ാണ് ഞാൻ ഈ പഠനത്തിൽ തിരഞ്ഞെടുക്കപ്പെട്ടത്?

Ans: You are chosen because you are identified by our research group as one of the subject who comes under the included age group, health status and mental status. You, as a person who satisfies all the potential criteria are being positively selected for the study.

ഉത്തരം: ഈ ഗവേഷണ പഠനത്തിന് അനുയോജ്യമായ പ്രായപരിധിക്കുള്ളിലും ആരോഗ്യ നിലവാരത്തിനുള്ളിലും മാനസിക ഘടനക്കുള്ളിലും താങ്കൾ ഉൾപ്പെടുന്നു. ഈ ഗവേഷണ പഠനത്തിന്റെ അവിഭാജ്യ ഘടകങ്ങളിൽ ഒന്നായതുകൊ ുമാണ് താങ്കളെ ഞങ്ങളുടെ ഗവേഷക സംഘം തിരഞ്ഞെടുത്തത്.

Q 3) Do I have to take part?

ചോദ്യം 3) ഞാൻ ഈ പഠനത്തിൽ പങ്കെടുക്കേ ആവശ്യകത ഉ ാ?

Ans: It is up to you to decide whether or not to take part in the study. We have selected educated and knowledgeable subjects for this study as self-determination and understanding the purpose of the study are considered very essential facts for the successful conduct of the study. There is absolutely no compulsion or compulsion like requests to you to take part in the study. If you decide positively to be a part of this study you will be given a consent form to get signed by you. If you are not willing to continue as a part of the study you can quit the study with proper communication to the team of researchers and you may not be taxed at any cost.

ഉത്തരം: ഈ പഠനത്തിൽ പങ്കെടുക്കണോ വേ യോ എന്നുള്ളത് താങ്കൾക്ക് സ്വയം തീരുമാനിക്കാവുന്നതാണ്. ഇതിൽ പങ്കെടുക്കുവാൻ ഒരു വ്യക്തിയേയും നിർബന്ധിക്കുവാൻ പാടുള്ളതല്ല. ഈ ഗവേഷണ പഠനത്തിൽ പങ്കെടുക്കുന്നവർ വിദ്യാഭ്യാസം ഉള്ളവരും സ്വയം തീരുമാനം എടുക്കുവാൻ പ്രാപ്തിയുള്ളവരും ആകണമെന്നത് നിർബന്ധം ആണ്. അതിനാൽ താങ്കളുടെ വ്യക്തി സ്വാതന്ത്ര്യത്തിനും യുക്തിബോധത്തിനും ഇത് വിട്ടുതന്നിരിക്കുന്നു. ഇത് സ്വയം ബോധ്യപ്പെട്ടാൽ മാത്രമേ നിങ്ങൾ സമ്മതപത്രം നല്കേ തുള്ളു. ഏത് സമയത്തും നിങ്ങളുടെ ആശങ്കകൾക്ക് ഉത്തരം നല്കുവാൻ ഞങ്ങൾ ബാധ്യസ്ഥരാണ്. ആയതിനാൽ നിങ്ങളുടെ വ്യാകുലതകൾ ഞങ്ങളെ അറിയിക്കാവുന്നതുമാണ്. ഏതെങ്കിലും സാഹചര്യത്തിൽ നിങ്ങൾക്ക് ഈ പഠനത്തിൽ തുടരുവാൻ താല്പര്യമില്ലാത്ത പക്ഷം യാതൊരു വിധ പിഴയും അടയ്ക്കാതെ തന്നെ താങ്കൾക്ക് ഇതിൽ നിന്നും പിന്മാറാവുന്നതാണ്.

Q 4) What will happen to me if I take part?

ചോദ്യം 4) ഞാൻ ഇതിൽ പങ്കെടുക്കുകയാണെങ്കിൽ എനിക്ക് എന്ത് സംഭവിക്കാം?

Ans: No harm will happen to you for any reason as it is a purely natural preparation. You can also expect the medicinal or the nutritional benefits of the product. You will be given the product totally free during the course of the trial and you may be advised to do laboratory test as per the protocol. At the end of the study period you may be asked to carry out certain tests and assessment measures to completely evaluate the medicinal and nutritional benefits of the product.

ഉത്തരം: താങ്കൾക്ക് ശാരീരികവും മാനസികവുമായ യാതൊരു ബുദ്ധിമുട്ടും ഉ ാവുകയില്ല. പ്രകൃതിദത്തമായ ഔഷധങ്ങളാൽ നിർമ്മിച്ചിട്ടുള്ള ഈ ഉല്പന്നം കഴിച്ചാൽ നിങ്ങൾക്ക് ഇതിന്റെ പോഷക ഗുണവും ഔഷധമൂല്യവും കൊ ുള്ള നേട്ടങ്ങൾ മാത്രമേ ഉ ാവുകയുള്ളു. ഈ ഗവേഷണ കാലാവധിയിൽ ഉടനീളം ഈ ഉല്പന്നം തികച്ചും സൗജന്യമായി നല്കുന്നതാണ്. പഠനത്തിനാവശ്യമായ പരിശോധനകൾക്ക് നിങ്ങൾ വിധേയരാവേ ി വരും. പഠന കാലാവധി കഴിഞ്ഞ ശേഷം ഈ ഉല്പന്നത്തിന്റെ പോഷകജനകവും ഔഷധാത്മകവുമായ മൂല്യങ്ങൾ വിലയിരുത്തുന്നതിനായി കുറച്ച് പരിശോധനകൾക്ക് താങ്കൾ വിധേയനാകേ ി വരും.

Q 5) What will happen to me if I don't take part?

ചോദ്യം 5) ഈ പഠനത്തിൽ പങ്കെടുത്തില്ലെങ്കിൽ എനിക്ക് എന്ത് സംഭവിക്കാം?

Ans: There will not be any problem if you don't take part in the study other than that you may not enjoy the medicinal or nutritional benefits of the product. It is all upto your 100% willingness to participate in the study. If you have any fear or doubt you need not take part in the study. "Diabeteaze" is a new product which is on clinical trial to find its nutritional and medicinal efficacy which might be effective for you as well, if you participate willingly.

ഉത്തരം: താങ്കൾ നിർബന്ധമായും ഈ പഠനത്തിൽ പങ്കെടുക്കെ തില്ല. മനസ്സിന്റെ പൂർണ്ണ പിന്തുണയുെ ങ്കിൽ മാത്രം താങ്കൾ ഇതിൽ പങ്കെടുത്താൽ മതിയാകും. അതല്ലാത്ത പക്ഷം താങ്കൾക്ക് പിന്മാറാവുന്നതാണ്. **"ഡയബറ്റീസ്"** എന്ന ഈ പുതിയ ഉല്പ്പന്നത്തിന്റെ പോഷകാത്മകവും ഔഷധാത്മകവുമായ ഗുണങ്ങൾ വിലയിരുത്തുന്ന ഈ പഠനത്തിൽ താങ്കൾ പങ്കാളി ആവുകയാണെങ്കിൽ താങ്കൾക്ക് അതിന്റെ ഗുണങ്ങൾ പ്രയോജനപ്പെടുത്തുവാൻ സാധിക്കും.

Q 6) What will I have to do?

ചോദ്യം 6) ഞാൻ ഈ പഠനത്തിൽ എങ്ങനെയെല്ലാം സഹകരിക്കണം?

Ans: Your sincere cooperation and unbiased observation of its effects are required for the study. If you wish to take part in the study at your own decision you have to discuss your health related issues, allergies, history of diabetes and other associated ailments with our team and answer some of the basic questions. All the information passed to us will be kept confidential and will be used only for the findings of the study and its support. If you are willing to use the product you have to follow the advice given by our team and should keep using this product as advised. You have to conduct certain lab investigations as and when suggested by our team. You are free to ask any doubt related to the study at any point of time and we are liable to answer it.

ഉത്തരം: ഈ പഠനത്തിന് വേ ി നിങ്ങളുടെ ആത്മാർത്ഥമായ സഹകരണവും, ഉല്പ്പന്നത്തിന്റെ ഫലങ്ങളെക്കുറിച്ചുള്ള പക്ഷാപാതമില്ലാത്ത നിരീക്ഷണവുമാണ് ഞങ്ങൾ പ്രതീക്ഷിക്കുന്നത്. ഈ ഗവേഷണ പഠനത്തോടൊപ്പം സഹകരിക്കുവാൻ തീരുമാനിക്കുന്ന പക്ഷം താങ്കളുടെ ആരോഗ്യ സംബന്ധമായ പ്രശ്നങ്ങൾ, അലർജി, ഡയബറ്റീസ് മൂലമു ാകന്ന മറ്റ് അനുബന്ധ പ്രശ്നങ്ങൾ എന്നിവയെക്കുറിച്ച് താങ്കൾക്ക് ഞങ്ങളോട് വിശദീകരിക്കേ ി വരാം. അതിനെ സംബന്ധിക്കുന്ന അനിവാര്യമായ ചോദ്യങ്ങൾക്ക് താങ്കൾ ഉത്തരം നല്കേ തായി വരും. ഈ വിവരങ്ങളെല്ലാം പഠനത്തിന്റെ നിരീക്ഷണത്തിനു വേ ി മാത്രമായിരിക്കും ഉപയോഗിക്കുകയെന്നും, അതീവ രഹസ്യമായി തന്നെ സൂക്ഷിക്കുമെന്നും താങ്കൾക്ക് ഉറപ്പ് നല്കുന്നു. ഇതിനോട് യോജിക്കുന്നുവെങ്കിൽ താങ്കൾ ഈ ഗവേഷണ പഠന കാലവധി തീരും വരെ ഞങ്ങളുടെ ടീം അംഗങ്ങളുടെ നിർദ്ദേശപ്രകാരം ഈ ഉല്പന്നം നിത്യേന ഉപയോഗിക്കുകയും ചെയ്യേ താണ്. ഇതിനോടനുബന്ധിച്ച് നിങ്ങളുടെ രോഗത്തിന്റെ നിർണ്ണയത്തിന് ആവശ്യമായ ലബോറട്ടറി പരിശോധനകൾക്ക് താങ്കൾ സഹകരിക്കണം. പഠനവുമായി ബന്ധപ്പെട്ട് നിങ്ങൾക്ക് എന്ത് ആശങ്കയുെ ങ്കിലും അതിന് ഉത്തരം നല്കുവാൻ ഞങ്ങൾ ബാധ്യസ്ഥരാണ്.

Q 7) Will my participation in this study kept confidential?

ചോദ്യം 7) എന്റെ ആരോഗ്യ വിവരങ്ങൾ രഹസ്യമായി സൂക്ഷിക്കപ്പെടുമോ?

Ans: Yes, the information furnished by you for the purpose of the study will be confidential. The only purpose of your details is to assess the nutritional and medicinal benefits of the product and it is made clear to you that this product may be beneficial to all the subjects who are part of the study and who are willing to consume the same in future.

ഉത്തരം: അതെ, നിങ്ങളെക്കറിച്ചുള്ള എല്ലാ വിവരങ്ങളും രഹസ്യമായി തന്നെ സൂക്ഷിക്കും എന്ന് ഞങ്ങൾ ഉറപ്പ് നൽകുന്നു. ഈ ഉല്പ്പന്നം ഉപയോഗിക്കന്നവർക്കും ഉപയോഗിക്കുവാൻ ഉദ്ദേശിക്കുന്നവർക്കും ഉ ാകുന്ന പോഷകാത്മകവും ഔഷധപരവുമായ നേട്ടങ്ങളെ വിലയിരുത്തുക എന്നത് മാത്രമാണ് ഈ പഠനം കൊ ് ഉദ്ദേശിക്കുന്നത്.

Q 8) What will happen to the results of the research study?

ചോദ്യം 8) ഈ ഗവേഷണ പഠന ഫലങ്ങൾ എന്തിനായി ഉപയോഗിക്കപ്പെടുന്നു?

Ans: We will utilize the study results for assessing the benefits and the other outcomes. It will help to identify the various medicinal and nutritional uses of the product and to communicate the same to the world.

ഉത്തരം: ഈ പഠനത്തിന്റെ നിരീക്ഷണങ്ങൾ ഉപയോഗിച്ച് ഈ ഉല്പന്നത്തിന്റെ പ്രയോജനവും അനന്തര ഫലങ്ങളും മനസിലാക്കാവുന്നതാണ്. ഇതിന്റെ പോഷണ ഗുണങ്ങളും ഔഷധമൂല്യവും ക ത്താനും അത് പ്രയോജനപ്പെടുത്തുവാൻ സമൂഹത്തിനോട് ആശയവിനിമയം നടത്തുവാനും സാധിക്കും.

Q 9) How long I have to be a participant in the study?

ചോദ്യം 9) എത്ര നാൾ ഞാൻ ഈ പഠനത്തിനായി സഹകരിക്കേ ി വരും?

Ans: As we are conducting the study for 6 months we recommend you to take part in the study throughout this period. Please remember that you can opt to go out of the study at any time without being taxed.

ഉത്തരം: പ്രാരംഭികമായി ഈ പഠനം 6 മാസം നടത്താൻ ഉദ്ദേശിക്കുന്നതിനാൽ ഈ കാലയളവിൽ ഉടനീളം നിങ്ങളുടെ സഹകരണം ഞങ്ങൾ പ്രതീക്ഷിക്കുന്നു.

Q 10) Is there any compensation or treatments available for me in case of study related injury?

ചോദ്യം 10) പഠനത്തോട് അനുബന്ധിച്ച് എന്തെങ്കിലും പാർശ്വഫലങ്ങൾ ഉ ായാൽ അതിനുള്ള നഷ്ടപരിഹാരമോ ചികിൽസയോ ലഭ്യമാകുമോ?

Ans: There will not be any injury as it is a purely herbal neutraceutical preparation. However if you want you may be given an insurance coverage during the study period.

ഉത്തരം: ഇതൊരു സസ്യജന്യമായ ഉല്പന്നം ആയതിനാൽ ഒരു പാർശ്വഫലങ്ങളും ഉ ാവാൻ സാധ്യത ഇല്ല. എന്നിരുന്നാൽ തന്നെയും നിങ്ങൾക്ക് ഈ പഠന കാലാവധിയിൽ ആവശ്യമെങ്കിൽ ഇൻഷുറൻസ് കവറേജ് ഉപയോഗപ്പെടുത്താവുന്നതാണ്.

Q 11) Who has reviewed the study?

ചോദ്യം 11) ആരാണ് ഈ പഠനം അവലോകനം ചെയ്യുന്നത്?

Ans: A team of well qualified professionals like doctors, pharmaceutical scientists and researchers are leading the study in a most scientific and professional manner. An independent ethics committee has been constituted with leading doctor, MLA, Scientist, theologist, social workers and socially accepted personalities. This committee is registered with the Drugs Controller of India (CDSCO) and the study will be overseen by the ethics committee.

ഉത്തരം: പ്രഗത്ഭരായ ഡോക്ടർമാരും ഗവേഷകരും അടങ്ങുന്ന ഒരു വിദഗ്ധ സംഘം ശാസ്ത്രീയവും നൂതനവും സുതാര്യവുമായ രീതിയിൽ പഠനം കൈകാര്യം ചെയ്യുന്നതായിരിക്കും. മാത്രമല്ല ഈ പഠനത്തിനായി വിദഗ്ധ ഡോക്ടർമാരും ഗവേഷകരും, എം.എൽ.എ യും മറ്റ് ബന്ധപ്പെട്ട അധികാരികളും അടങ്ങുന്ന ഒരു സ്വതന്ത്ര എത്തിക്സ് കമ്മിറ്റി രൂപീകരിച്ച് ഡ്രഗ് കൺ ട്രോളർ ഓഫ് ഇൻഡ്യ (സി.ഡി.എസ്.സി.ഒ) യിൽ രജിസ്റ്റർ ചെയ്തിട്ടു ്. ഈ എത്തിക്കൽ കമ്മിറ്റിയുടെ വിശദവിവരങ്ങൾ പങ്കജകസ്തുരി ഹെർബൽ റിസർച്ച് ഫൗ േഷന്റെ വെബ്സൈറ്റിൽ ലഭ്യമാണ്. ഈ കമ്മിറ്റിയുടെ നിരീക്ഷണങ്ങൾക്ക് വിധേയമായാണ് ഈ ഗവേഷണ പഠനം നടത്തുന്നത്.

വിശദവിവരങ്ങൾക്ക് ബന്ധപ്പെടുക: **ഡോ. ഷാൻ ശശിധരൻ, ട്രയൽ മോണിറ്റർ.**

ഈ വിവരങ്ങൾ വായിച്ചു മനസിലാക്കിയതിന് നന്ദി.

APPENDIX – 2/ അപ്പന്റിക്സ് – 2

INFORMED CONSENT FORM/ സമ്മതപത്രം

Study Title/ പ്രൊജക്റ്റിന്റെ പേര്: A RANDOMIZED STUDY TO EVALUATE THE EFFECT OF DIABETEAZE POWDER IN NUTRITIVE MANAGEMENT OF TYPE II DIABETES MELLITUS.

Study Number / പ്രോജക്ട് നമ്പർ :

Study site / area / location / ഗവേഷണ സ്ഥലം/തലൂക്ക്/വില്ലേജ് :

Subject initials: / ഉപഭോക്താവിന്റെ ഒപ്പ് :

Subjects name: ഉപഭോക്താവിന്റെ പേര് :

Date of birth / age / ജനന തിയതി/വയസ്സ് :

Educational qualifications / വിദ്യാഭ്യാസ യോഗ്യത :

I, ……………………………………………………………………………………. Am exercising my free power of choice, and am hereby give my consent to be included as a subject for the clinical trial titled "**A randomized study to evaluate the effect of DiabetEaze Powder in nutritive management of type II diabetes mellitus**"

……………………………………………………………………………………………… എന്ന ഞാൻ എന്റെ സ്വബോധത്തോടെയും താല്പര്യത്തോടെയും ആണ് ഈ പ്രോജക്ടിന്റെ ഭാഗമാകുന്നത് എന്ന് സാക്ഷ്യപ്പെടുത്തുന്നു.

If you agree to the following: - Please (✓) in the box provided at the left side of each undertaking.

താങ്കൾ ചുവടെ പറയുന്ന കാര്യങ്ങളോട് അനകൂലിക്കുന്നു എങ്കിൽ അതിന് നേരെ ഉള്ള ബോക്സിൽ (✓) രേഖപ്പെടുത്തുക.

☐ 1. I confirm that I have read and understood the consumer information sheet and signed it at my own choice. I am given full freedom and opportunity to ask questions and clarify them.

ഉപഭോതൃ വിവരപ്പട്ടികയിലെ നിയമാവലികൾ ഞാൻ വായിച്ച് ബോധ്യപ്പെട്ട് എന്റെ സംശയങ്ങൾ എല്ലാം ദൂരീകരിച്ചതിന് ശേഷമാണ് ഈ സാക്ഷ്യപത്രത്തിൽ ഒപ്പിടുന്നത്.

☐ 2. I understand that this product is manufactured out of pure herbal ingredients. It is a dietary supplement expected to have medicinal and nutritional effect because of the ingredients in it.

ഈ ഔഷധം പ്രകൃതിദത്തമായ സസ്യങ്ങളാൽ നിർമ്മിക്കപ്പെട്ടതും പോഷകങ്ങളും ഔഷധഗുണങ്ങളും ഉള്ള ഒരു ഉല്പന്നം ആണെന്നും എനിക്ക് ബോധ്യപ്പെട്ടിരിക്കുന്നു.

☐ 3. I understand that I am included in the study / selected for the study as I am inside the prescribed age limit, mental and physical status and I am planning to take the product as prescribed by the doctor / pharmacological scientist / researcher.

ഈ ഗവേഷണത്തിന് ഉതകുന്ന പ്രായപരിധിയിൽപെടുന്നതിനാലും, ശാരീരിക, മാനസിക തലങ്ങളിൽ ഈ പഠനം അനകൂലമാകും എന്നതിനാലും ആണ് ഈ ഗവേഷണ സംഘം നിർദ്ദേശിക്കുന്ന തരത്തിൽ ഈ ഉല്പന്നം സേവിക്കാൻ തിരഞ്ഞെടുക്കപ്പെട്ടത് എന്ന് ഞാൻ മനസ്സിലാക്കുന്നു.

☐ 4. I am fully aware that I may need to give blood samples or get my blood samples tested in approved clinical laboratories on certain days during the study period for the purpose of estimation / evaluation / analysis of certain glycemic values and associated indices.

ഈ പഠനകാലത്ത് ഗവേഷണ സംഘം നിർദ്ദേശിക്കുന്ന ലബോറട്ടറി പരിശോധനകൾക്ക് വിധേയൻ ആകേ ി വരുമെന്നും പഠനത്തിന്റെ വിലയിരുത്തലിന് അത് അത്യാവശ്യമാണെന്നും ഞാൻ മനസ്സിലാക്കുന്നു.

☐ 5. I also understand that through in house pilot studies on **DiabetEaze** have proven effects on nutrition and overall health of Type II diabetes mellitus patients.

''ഡയബറ്റീസ്'' എന്ന ഈ ഉല്പന്നത്തിന് ടൈപ്പ് 2 ഡയബറ്റീസ് രോഗികളിൽ പോഷകാത്മകവും ഔഷധപരവുമായ മാറ്റങ്ങൾ വരുത്തുവാൻ സാധിക്കുന്നു എന്ന് സ്ഥാപനത്തിൽ നടത്തിയ പരീക്ഷണങ്ങളിലൂടെ ബോധ്യപ്പെട്ടതായി ഞാൻ മനസ്സിലാക്കുന്നു.

☐ 6. I understand that **DiabetEaze** is purely a herbal product and is not having any serious side effects that can affect my health condition in any way.

''**ഡയബറ്റീസ്**'' എന്നത് തികച്ചും പ്രകൃതിദത്തമായ ഒരു ഉല്പന്നം ആണെന്നും ഇത് സേവിക്കുന്നത് കൊ ് എനിക്ക് ആരോഗ്യപരമായ യാതൊരു ബുദ്ധിമുട്ടും ഉ ാവുകയില്ലെന്നും ഞാൻ മനസ്സിലാക്കുന്നു.

☐ 7. I understand the medicinal and nutritional value of herbal ingredients used and the importance of including this herbal product in my daily diet.

ഈ ഉല്പന്നത്തിൽ അടങ്ങിയിരിക്കുന്ന ഓരോ ചേരുവകളുടേയും ഔഷധ പോഷക മൂല്യങ്ങൾ ഞാൻ മനസ്സിലാക്കി എന്നും എന്റെ ദിവസേനയുള്ള ഭക്ഷണക്രമത്തിൽ ഇത് ഉൾപ്പെടുത്തിയതിന്റെ ആവശ്യകതയെ കുറിച്ചും ഞാൻ മനസ്സിലാക്കുന്നു.

☐ 8. I am given a full information / explanation by the supervising health care professional team regarding the nature, purpose, likely duration of the study and benefits.

ഈ പഠന പക്രിയയുടെ സ്വഭാവവും ആവശ്യകതയും സമയപരിധിയും വരുംവരായ്കകളും ഗവേഷണ സംഘം വ്യക്തമായി പറഞ്ഞ് എനിക്ക് ബോധ്യപ്പെടുത്തിയിട്ടു ്.

☐ 9. I have been given the opportunity to question on all the aspects of the study including my own doubts and I have understood the advice and information given to me to the best of my satisfaction.

ഈ പഠനത്തോട് അനബന്ധിച്ചുള്ള എന്റെ എല്ലാവിധ സംശയങ്ങൾക്കും കൃത്യമായ ഉത്തരം നൽകാൻ ഗവേഷണ സംഘത്തിന് സാധിച്ചു എന്ന് ഞാൻ സാക്ഷ്യപ്പെടുത്തുന്നു.

☐ 10. I have not taken part in any clinical trial study for the past 6 monthsand particularly for diabetes during last 1 year.

ഈ കഴിഞ്ഞ 6 മാസത്തിനള്ളിൽ ഒരു രോഗ ഗവേഷണത്തിനും ഞാൻ പങ്കാളി ആയിരുന്നില്ല എന്നും കഴിഞ്ഞ ഒരു വർഷത്തിനിടക്ക് പ്രസ്തുത ഗവേഷണ വിഷയമായ ഡയബറ്റീസ് പഠനത്തിന്റേയും ഭാഗമായിരുന്നില്ല എന്നും സാക്ഷ്യപ്പെടുത്തുന്നു.

☐ 11. I am also aware of my right to opt out of the study at any time without being taxed.

എനിക്ക് താല്പര്യമില്ലാത്ത പക്ഷം ഏത് സമയത്തും ഒരു പിഴയും അടക്കാതെ തന്നെ ഈ പഠനത്തിൽ നിന്നും പിന്മാറുവാൻ സാധിക്കും എന്ന് എനിക്ക് ഉത്തമ ബോധ്യമു ്.

☐ 12. I have been informed regarding whom to contact for the study related queries and questions.

ഈ പഠനവുമായി ബന്ധപ്പെട്ട എന്റെ ആശങ്കകൾ ദൂരീകരിക്കാൻ ആരുമായി ബന്ധപ്പെടണമെന്ന് എനിക്ക് പൂർണ്ണ ബോധ്യമു ്.

☐ 13. I understand that the team of clinical experts, ethics committee and the regulatory authorities will not need my permission to look at my health records in respect of current research study to assess the nutritive and therapeutic efficacy of diabeteaze.

ഈ പഠനത്തിന്റെ അടിസ്ഥാന ഉല്പന്നത്തിന്റെ പോഷക ഔഷധ ഗുണങ്ങൾ വിലയിരുത്താൻ എന്റെ ആരോഗ്യ സംബന്ധമായ രേഖകൾ പരിശോധിക്കാൻ ഈ ഗവേഷണ സംഘത്തിന് അവകാശമുെ ന്ന് ഞാൻ മനസ്സിലാക്കുന്നു.

☐ 14. I understand that my clinical data, obtained during the trial or within six months after study enrollment, will be shared for publication in a scientific journal or magazine. However, my confidential medical records, which reveal my identity, will remain private unless required by law.

ട്രയൽ വേളയിലോ, പഠന എൻറോൾമെന്റിന് ശേഷം ആറുമാസത്തിനുള്ളിലോ ലഭിച്ച എന്റെ ക്ലിനിക്കൽ ഡാറ്റ, ഒരു സയന്റിഫിക് ജേർണലിലോ മാസികയിലോ പ്രസിദ്ധീകരിക്കുന്നതിനായി പങ്കിടുമെന്ന് ഞാൻ മനസ്സിലാക്കുന്നു. എന്നിരുന്നാലും, എന്റെ ഐഡന്റിറ്റി വെളിപ്പെടുത്തുന്ന രഹസ്യാത്മക മെഡിക്കൽ രേഖകൾ, നിയമം ആവശ്യപ്പെടുന്നില്ലെങ്കിൽ സ്വകാര്യമായി തുടരും.

Signature or thumb impression of the subject

ഉപഭോക്താവിന്റെ ഒപ്പ്/ വിരലടയാളം :

Date. /തിയതി :

Study Signature of the study investigator/ഗവേഷകന്റെ ഒപ്പ് :

Investigator’s name. /ഗവേഷകന്റെ പേര് :

Date /തിയതി :
